# Flurbiprofen loaded ethosomes - transdermal delivery of anti-inflammatory effect in rat model

**DOI:** 10.1186/s12944-019-1064-x

**Published:** 2019-06-07

**Authors:** Sarvesh Paliwal, Amita Tilak, Jaiprakash Sharma, Vivek Dave, Swapnil Sharma, Renubala Yadav, Saraswati Patel, Kanika Verma, Kajal Tak

**Affiliations:** 1 Department of Pharmacy, Banasthali Vidyapith, Banasthali, Rajasthan 304022 India; 2 Department of Pharmacy, SMS Medical College, Banasthali, Rajasthan India

**Keywords:** Ethosomes, Flurbiprofen, Transdermal delivery

## Abstract

**Background:**

Ethosomes have been widely used in Transdermal Drug Delivery System (TDDS) as they increase the permeation of drug across the skin.

**Methods:**

Flurbiprofen loaded vesicular ethosomes were formulated, optimized and characterized for particle size, entrapment efficiency, poly dispersive index (PDI), microscopy using Atomic force microscopy (AFM), Scanning electron microscope (SEM) and Transmission electron microscopy (TEM) and Interaction of drug and excipients were studied using Fourier transform infra-red (FTIR) spectroscopy, Differential scanning colorimetry (DSC), Thermo gravimetric analysis (TGA). Further, ethosomal formulations of flurbiprofen were evaluated for stability study of three months and in vitro drug permeation study was carried out using albino rat skin. In addition, skin irritation test was evaluated by Draize test and in vivo study of prepared formulation was examined through paw edema assay by inducing carrageenan and cold plate method.

**Results:**

Amongst all formulations, EF5 formulation exhibited ideal surface morphology, with maximum entrapment efficiency (95%) with optimal excipient concentration i.e. 200 mg phospholipid and 35% ethanol. The ideal vesicle size was achieved as 162.2 ± 2 nm, with zeta potential − 48.14 ± 1.4 mV with the PDI of 0.341. In-vitro permeation study shows a release of 82.56 ± 2.11 g/cm^2^ in 24 h and transdermal flux was found as 226.1 μg/cm^2^/h. Cold plate test indicates that the formulation EF5 showed a marked analgesic activity and Carrageenan induced paw edema test indicates that the formulation EF5 inhibits the increase in paw edema. Ethosomal suspension at 4 °C showed maximum stability.

**Conclusions:**

The overall study concluded that this ethosomal approach offers a new delivery system for sustained and targeted delivery for flurbiprofen.

**Electronic supplementary material:**

The online version of this article (10.1186/s12944-019-1064-x) contains supplementary material, which is available to authorized users.

## Background

Transdermal drug delivery system (TDDS) offers many advantages in the modern drug therapy and uses skin as possible route for drug administration to attain systemic effect. Transdermal patches regulate the delivery of drugs by engaging a suitable combination of hydrophilic and lipophilic polymers. Ethosomes has been reported as the novel innovative passive, noninvasive delivery carriers for lipid based system with attractive features.

Ethosomes carriers were first reported by Touitou and her colleagues in 2000. These are spongy, soft and malleable vesicles modified for improved drug delivery of active agents. Generally, ethosomes which are phospholipid vesicular system have lipid bilayers like liposomes. Ethosomes are composed of phospholipids, high concentration of ethanol and water whereas liposomes composition uses cholesterol instead of ethanol. Ethanol as an important adjuvant exhibits impressive transdermal permeability enhancing properties and also results in softening of ethosomal lipid bilayer. Therefore, the presence of ethanol in ethosomes formulation offers rapid permeation of entrapped drug molecules through skin. Additionally, it results in steady state flux (approximately 1 mg/cm^2^/h) across skin in comparison to liposomes which remain confined to the upper layer of stratum corneum [1]. The mechanism for enhanced pervasion of drug into deeper skin layers from ethosomes is still not well understood. However, the enhanced permeability of ethosome carriers is possibly related to the collective mechanism between high concentration of ethanol, phospholipids vesicles and skin lipids. Ethanol act together with lipid molecules in the polar head group region ensuing in increased fluidity and resulting in increased membrane permeability. Ethosomes have been considered as improved form of liposomes [2]. In different studies ethosomes have been used widely as a carrier system for topical administration. Various reports have reported that ethosomes have increased drug deliveries across skin both at in vitro and in vivo level, such as Ketoprofen, Testosterone, Cannabidiol, Buspirone hydrochloride, Erythromycin, Ammonium glycyrrhizinate, Ibuprofen, Benzocaine, Fluconazole, Finasteride, Lamivudine and 5-aminolevulinic acid. Interestingly, ethosomes preparation does not require special equipment’s and thus is considered easy to formulate. Discovery of ethosomes has started a new revolution in vesicular research for transdermal drug delivery.

Flurbiprofen, a non-steroidal anti-inflammatory drug which is phenylalkanoic acid derivative exhibits antipyretic, anti-inflammatory and analgesic activities. Oral formulations of flurbiprofen are indicated in acute or chronic symptomatic treatment of rheumatoid arthritis, osteoarthritis and anklylosing spondylitis. It is also useful in pain reduction concomitant with dysmenorrhea and in bursitis, tendonitis and soft tissue trauma. Flurbiprofen is the most potent non-steroidal anti-inflammatory agent which reversibly inhibits cyclooxygenase (COX), an enzyme accountable for metabolism of arachidonic acid. The cyclooxygenase (COX) inhibition decreases prostaglandin production, preferably the highly pruritic PGE2 and PEG2 [3].

In view of this, in the present work, we have entrapped flurbiprofen in the lipid bilayers of ethosomes to develop sustained transdermal delivery of ethosomal gel of flurbiprofen and evaluated its performance using in vitro and in vivo models.

## Methods

### Materials

Flurbiprofen was purchased as a gift sample from Lupin Research Park, Pune, India. Soya Phosphatidylcholine, ethanol, chloroform, propylene glycol, isopropyl alcohol and methanol were purchased from Sigma-Aldrich chemicals, Germany. Carbopol 934 K was received from Himedia laboratory Mumbai, India. All other chemicals used during the experiment were of analytical grade.

## Methods

### Preparation of flurbiprofen loaded ethosomes

Flurbiprofen loaded ethosomes were prepared through the method discussed by Touitou (2000). The formulations for ethosomal system of flurbiprofen were prepared using 100 mg, 200 mg, 300 mg phospholipids, 30, 35, 40% w/w of ethanol, 250 mg flurbiprofen (API), 1% propylene glycol (PEG) and water (%) to q.s., depicted in Table 1. Initially, ethanol and propylene glycol was used to dissolve the drug and phospholipid and the solution was allowed to heat at temperature of 30 °C on water bath. Further distilled water was added steadily as a fine stream to the solution with continuous stirring using magnetic stirrer (Remi equipment, Mumbai) at the speed of 700 rpm in a closed vessel. Then prepared solution was maintained at the temperature of 4 °C. Afterwards sonication was done by the means of probe sonicator for 3 cycles of 5 min (with 5 min’ rest between the cycles) [4]. Same protocol was followed for the Gliclazide ethosomal preparation by thin film hydration method which shows an improved choice for sustained release of drug through topical drug delivery [5].

**Table 1:** Composition of flurbiprofen containing ethosomes

Composition	EF1	EF2	EF3	EF4	EF5	EF6	EF7	EF8	EF9
Drug (Flurbiprofen) mg	250	250	250	250	250	250	250	250	250
Soya lecithin (mg)	100	100	100	200	200	200	300	300	300
Ethanol (%)	30	35	40	30	35	40	30	35	40
Propylene glycol (%)	1	1	1	1	1	1	1	1	1
Water (%)	q.s	q.s	q.s	q.s	q.s	q.s	q.s	q.s	q.s

### Flurbiprofen loaded ethosomal gel

Carbopol 934 K 0.75% w/v was immersed in small amount of water for an hour. With continuous stirring, 20 ml of ethosomal suspension containing flurbiprofen (250 mg) was added to the swollen polymer. Stirring was continued at the speed of 700 rpm in a closed vessel to achieve homogeneous ethosomal gel consistency at temperature of 30 °C. Triethanolamine (TEA) was added to neutralize the pH with slow stirring until the gel was formed. The pH of the semisolid ethosomal gel formulations was measured by using pH meter [6].

### Physicochemical characterization of flurbiprofen loaded ethosomal system

#### Particle size, zeta potential and Polydispersive index (PDI)

The characteristics like average size, particle size distribution and surface charge of the formulated ethosomes were determined by dynamic light scattering (DLS) using Malvern Zeta sizer Nano ZS (Malvern Instruments, UK) at room temperature. Ethosomal formulations were dispersed in HPLC water and an instrument was set at an angle of 90° with a medium viscosity of 0.8862 and refractive index 1.361. The particle size distribution, poly dispersibility index and zeta potential were determined; all the measurements were made in triplicates [6].

#### Morphology of Flurbiprofen loaded ethosomes by TEM, SEM, and AFM

The morphology of prepared flurbiprofen loaded ethosomes was studied using Transmission electron microscopy (TEM) (AIIMS, Delhi) operated at 200 kV at a magnification of 9900x. 20 μl of the Flurbiprofen loaded ethosomal suspension was diluted with millipore water and was allowed to stain with 2% w/v phosphotungstic acid for about 30 s. Sample was placed on coated copper grid and was allowed to dry. For every sample, two grids were prepared and randomly viewed [7].

Scanning electron microscopy (SEM) was performed using lyophilized sample fixed on carbon tape and palladium coating was done to provide the conductivity to the sample during the electron bombardment. Imaging was done using SEM (Banasthali Vidyapith, Rajasthan, India) performed at 20 kV at magnification of 3300x, 19,000x [8].

For atomic force microscopy (AFM) analysis, 1 ml of ethosomal suspension was carefully placed on a neatly sliced mica sheet and left for incubation of five minutes. From the surface of mica sheet, unbound ethosomes were removed by rinsing with deionized water. Sample was air dried at normal room temperature and was scanned using Advance Integrated Scanning tool for Nano Technology (AIST-NT) Model: Smart SPM 1000, (NIIFP, Russia) in tapping mode. Every sample was scanned, at a resolution of 5 μm × 5 μm and images were observed simultaneously by showing height, amplitude and phase signal of the cantilever in the trace direction.

#### Attenuated total reflection fourier transforms infra-red spectroscopy (ATR-FTIR)

Infrared spectra of pure drug (flurbiprofen), flurbiprofen loaded ethosomes, physical mixture soya lecithin and optimized ethosomal formulation were scanned by Bruker EQUINOX 55 FTIR spectrophotometer furnished by a liquid nitrogen cooled mercury cadmium telluride (MCT) detector at resolution of 2 cm^_1^. The internal reflection element (IRE) is made up of diamond with an incidence angle of 45^o^, the speed of scans rate was 32, 21 resolutions leading to one internal reflection. An innovative attenuated total reflection (ATR) modification was applied to all spectrums, and the region of graph line was from 4000 to 400 cm^_1^ using ATR-FTIR Opus software.

#### Raman spectroscopy

Raman spectra of the flurbiprofen loaded ethosomes were scanned with a spectrophotometric system (Thermo-scientific instrument (DxRxi), equipped with a OMINICxi-Analysis software. The 532 nm laser beam was used to collect the spectra with the laser power of 5-100 mW. A range of 125–4000 cm^− 1^ was selected to scan and obtain entire spectrum.

#### Differential scanning calorimetry (DSC)

Flurbiprofen, soya lecithin, and lyophilized formulation EF5, were used for DSC (Model: DSC 204 F1 PHOENIK NETEZCH) analysis. The samples (drug, lipid and lyophilized ethosomes) were crimped placed and placed on aluminium pan and were crimped. Further, sample was heated and nitrogen was flowed (30 ml/min) at a scanning rate of 5 °C/min from 25 °C to 300 °C. As a reference same quantity of indium on another aluminium pan was placed and scanned. For drug and drug loaded ethosomes, the range of temperature for heat flow was measured [9]. Afterwards, the scan were recorded and plotted on the graph, representing temperature and heat flow (w/g) on the X-axis and Y-axis respectively.

#### Thermo gravimetric analysis (TGA)

In order to study the various physicochemical properties, TGA studies of drug, lipid and lyophilized formulation were performed using TGA-4000 Perkin Elmer with Pyris Manager Software. First, crucible was weighed empty then sample was weighed in crucible and were kept back on the assembly which was made to run the sample. The scan was recorded and plotted graph showing percent weight loss on Y-axis and temperature on X-axis respectively [10].

#### Entrapment efficiency of ethosomes

The % entrapment efficiency (%EE) of formulated ethosomes was calculated by subtracting the amount of un-entrapped drug by total amount of drug taken. Flurbiprofen amount was analyzed using centrifugation method i.e. by centrifuging the formulations by means of ultracentrifuge (Remi) equipped with TLA-45 rotor at 14,000 rpm at 4 °C for 30 min. After first centrifugation supernatant was removed and re-centrifuged for 15 min at 14,000 rpm. The sum total of unentrapped flurbiprofen was examined using UV/Vis-spectrophotometer at 272 nm. Each sample was evaluated thrice [11, 12]. The percentage of encapsulated drug amount was calculated using the following formula:

$$ \mathrm{EE}\ \left(\%\right)=\left(\mathrm{Amount}\ \mathrm{of}\ \mathrm{the}\ \mathrm{drug}\ \mathrm{in}\ \mathrm{the}\ \mathrm{ethosomes}/\mathrm{Total}\ \mathrm{amount}\ \mathrm{of}\ \mathrm{drug}\ \mathrm{loaded}\ \mathrm{in}\mathrm{to}\ \mathrm{the}\ \mathrm{ethosomes}\right)\times \kern0.37em 100\%. $$

#### Viscosity, pH and Spreadability

The viscosity of the gel was observed by Brookfield viscometer (model LVDV II Pro) at 20 rpm of fixed speed, and pH was monitored by digital pH meter. For the spreadibility measurement, prepared ethosomal formulation (1 g) was sandwiched between two glass slides having 8 cm length [13]. Then different weight was tied from the pulley and the weight at which the glass slide move and the time required to pull the upper slide and further spreading of gel to the lower slide was measured. The measurement of length of displaced slide was taken in triplicate and spreadability was calculated using to the described formula:

$$ \mathrm{S}=\mathrm{M}\times \mathrm{L}/\mathrm{T} $$

Where, S represents the Spreadability of the gel, M is the weight knotted to the upper slide (g), L shows the distance moved by the slides (cm) and t is the time taken by the upper slide to roll down.

#### Skin irritation studies

Draize patch test was performed to examine the irritancy of plain flurbiprofen solution and optimized formulation in Albino rats. Using the intact skin of Albino rats the irritation potential of flurbiprofen and EF5 was studied. Before 24 h of the start of the assay, dorsal hair of the animal was shaved. Animals were allocated into three groups (1st group- no drug, 2nd group- flurbiprofen, 3rd group- optimized formulation) having six animals (*n* = 6) in every group. Further, 2 ml of flurbiprofen solution and EF5 were applied uniformly on the shaved skin of rats and any visible changes were observed at 24, 48, and 72 h and scored accordingly [14].

#### In vitro skin permeation study

To perform the in-vitro skin permeation test, male Albino Wistar rats (150–200 g) were used. Briefly, hair of dorsal region of rat were removed in an area of 2cm^2^ of the skin using hair removal cream [15] (Anne French; Wyeth Ltd., Hyderabad, India). Further, rats were scarified by high dose of anesthesia and dorsal hair free skin was isolated. Subsequently, subcutaneous fat and connective tissues were neatly trimmed using sharp scissor. Later, the skin was washed with physiological salt solution (PSS) and then by distilled water top and placed in refrigerator at 4 °C in PBS for further use [16]. The in vitro drug permeation from flurbiprofen loaded ethosomal formulation was determined using Franz’s diffusion cell technique, via rat skin considering it as semipermeable membrane. Digital micrometer was used to measure thickness of the skin which was found to be 2.8 ± 0.7 mm. Thereafter, skin was cautiously clamped with the Franz’s diffusion cell having effective permeation area (0.64 cm^2^) and receptor volume (10 ml) with use of phosphate buffer saline (pH 7.4). Accurately weighed 1 g of the Flurbiprofen loaded ethosomal gel and transferred it to donar compartment of the Franz’s diffusion cell, at a constant temperature i.e. 37 ± 0.5 °C was maintained with continuous stirring using magnetic stirrer (Expo India Ltd., Mumbai, India) at 200 rpm. At predetermined time intervals (0.5, 1, 2, 6, 8,…..12, 24 h.), samples were withdrawn and to maintain the sink condition, instantly same volume of blank receptor fluid was added to the receptor compartment. HPLC method was further used to analyze the drug concentration. The experiment was performed in triplicates and expressed as the Mean ± SD. For all ethosomal batches, in vitro skin permeation studies were accomplished [17]. All analysis was made in accordance with the standard institutional guiding principles duly proved by the Committee for the Purpose of Control and Supervision of Experiments on Animals (CPCSEA) of Banasthali University Animal Centre (Rajasthan, India).

### Data treatment

The flux of the formulated flurbiprofen loaded ethosomes was calculated through the Fick’s second law of diffusion, in which the total amount of drug (Q_t_) appearing in the receptor compartment in time t is expressed as:

1 $$ {\mathrm{Q}}_{\mathrm{t}}={\mathrm{AKLC}}_{\mathrm{o}}\left[\left({\mathrm{D}}_{\mathrm{t}}/{\mathrm{L}}^2\right)-\left(1/6\right)-\left(2/{\uppi}^2\right)-\upepsilon \left({\left(1-1\right)}^{\mathrm{n}}/{\mathrm{n}}^2\right)\exp \left({\mathrm{D}}^{\mathrm{n}}{2\uppi}^2\mathrm{t}/{\mathrm{L}}^2\right)\right] $$

Where,

A = effective diffusion area,

C_0_ = concentration of drug that remain constant in the vehicle,

D = diffusion coefficient,

L = thickness of the skin membrane and.

K = partition coefficient of the drug between membrane and the vehicle.

At steady state, Eq. (1) can represent as follows:

2 $$ \mathrm{Qt}={\mathrm{KLC}}_{\mathrm{O}}\left[\left({\mathrm{D}}_{\mathrm{t}}/{\mathrm{L}}^2\right)-\left(1/6\right)\right] $$

Thus, the flux (J) was calculated through the slope of the equation to show steady state position of the amount of the drug permeated through an area with respect to a given time t. Hence, from Eq. (2), the flux (J) can be expressed as follows:

3 $$ \mathrm{J}={\mathrm{C}}_0\mathrm{KD}/\mathrm{L}={\mathrm{C}}_0\mathrm{Kp} $$

Where, Kp is known the permeability coefficient [18].

#### In vivo activity all

animal experimental protocols were approved by Institutional Animal Ethical Committee (Ref. no. BU/3431/16–17) and conducted in accordance to the Committee for the Purpose of Control and Supervision of Experiments on Animals (CPCSEA) guidelines. Male Wistar rats (weighing 250–300 g) and mice (weighing 20–30 g), were employed for the experiments. The rats were procured from Lala Lajpat Rai Veterinary and Animal Sciences, Hisar, India. They were kept in polyacrylic cages and maintained under standard housing conditions of temperature (25 ± 2 °C) and humidity (60–65%) with 12-h light-dark cycles. They were acclimatized for one month and given ad libitum access to food (dry pellets) and water.

#### Cold plate test

Analgesic activity was assessed by measuring reaction time of rats at low temperature. Paw licking, jumping, defecation, urination, shivering was noted as latency of rats. Rats were divided in three different groups (*n* = 6) and received treatment as shown in experimental design (Table 2). Group I was subjected to Brugel® marketed preparation whereas animals of group II-III received different concentration of test formulations topically, respectively. For induction of pain, the rats were placed on cold plate (4 °C) and latencies of both forepaw licking (reaction time; cutoff time: 15–20 s) were measured for each animal using a stopwatch at a regular interval of 15 min till 1 h [19].

**Table 2:** Experimental Design (*n* = 6)

Group	Treatment
I	Brugel Gel
II	Etho 5 (10 mg/kg)
III	Etho 5 (20 mg/kg)

#### Carrageenan induced paw edema

Rats were divided in three groups (*n* = 6) and received treatment as shown in experimental design (Table 2). Acute edema was induced by injecting 0.1 ml of freshly prepared 1% solution of carrageenan in sub-plantar tissue of rat right hind paw. After 30 min of induction, Brugel® marketed preparation was applied to animals of group I whereas group II and III animals received topical application of test formulations (10 and 20 mg/kg) respectively. Reduction in edema was measured at every 15 min up to 2 h using plethysmometer. Percent change in paw volume was calculated and expressed as the swelling index [19].

#### Stability studies

To determine the drug retention ability by the formulated ethosomal vesicles, stability studies were performed for three months at different temperature. The stability of vesicles is the major problem in the formulation of ethosomes because of leaching and drug accumulation properties in the lipid layers. The optimized formulation EF5 was selected for the stability studies. Two batches of lyophilized ethosomes and ethosomal suspension were divided and kept in sealed vials (10 ml) at 4 °C/60 ± 5 RH (*n* = 3) and at 25 °C/60 ± 5 RH. As a function of time the sample were analyzed after 7, 15, 30, 60 and 90 days of storage (Table 3).

**Table 3:** Stability study of optimized formulation EF 5

Time (days)	Microscopic evaluation	%Encapsulation efficiency
Ethosomal suspension (EF 5)		
At 4 °C/60 ± 5RH (*n* = 3)		
Initial	Smooth spherical vesicles	95 ± 1
7	Smooth spherical vesicles	91.5 ± 2.3
15	Smooth spherical vesicles	88.4 ± 3.2
30	Smooth spherical vesicles	85.4 ± 1.2
60	Rough spherical vesicles	80.7 ± 3.3
90	Rough spherical vesicle	74.5 ± 1.4
At 25 °C/60 ± 5RH (*n* = 3)		
Initial	Smooth spherical vesicles	95 ± 1
7	Smooth spherical vesicles	89.5 ± 1.3
15	Smooth spherical vesicles	86 ± 3
30	Rough spherical vesicles	73.7 ± 1.8
60	Agglomerate	68.2 ± 2
90	Agglomerate	54.5 ± 1.4
Lyophilized ethosomal suspension (EF5)		
At 4 °C/60 ± 5RH (*n* = 3)		
Initial	Smooth spherical vesicles	95 ± 1
7	Smooth spherical vesicles	93.5 ± 1.2
15	Smooth spherical vesicles	89.5 ± 3.1
30	Smooth spherical vesicles	86.43 ± 2
60	Rough spherical vesicles	80.3 ± 1.2
90	Rough spherical vesicles	78.3 ± 1.9
At 25 °C/60 ± 5RH (*n* = 3)		
Initial	Smooth spherical vesicles	95 ± 1
7	Smooth spherical vesicles	88.4 ± 1
15	Rough spherical vesicles	81.7 ± 2.4
30	Rough spherical vesicles	77.9 ± 3
60	Rough spherical vesicles	71 ± 2.5
90	Agglomerate	65.5 ± 2

#### Statistical analysis

Statistical analysis results were calculated using a computer based, Graph Pad Prism version 5.1 software program. One-way ANOVA was used to statistically estimate the data with Tukey Posthoc test. *P* values less than 0.05 (*p* < 0.05) considered statistically significant. All values were conveyed as the mean value ± standard deviation.

## Results and discussion

### Ethosomal formulation

Various ethosomal formulations have been reported for topical drug delivery for multiple diseases. Different formulations of flurbiprofen loaded ethosomes were formulated by varying the concentration of ethanol and soya lecithin (Table 1). Formulation EF1 to EF3 and formulation EF4 to EF6 contained constant amount of soya lecithin i.e. 100 mg and 200 mg respectively with varying in concentration of ethanol 30, 35 & 40% respectively. Similarly, formulation EF7 to EF9 had highest concentration of soya lecithin i.e. 300 mg with varying in concentration of ethanol.

#### Particle size, zeta potential, polydispersity index (PDI)

Dynamic light scattering (DLS) technique was used to measure the particle size and polydispersity index (PDI), shown in Table 4. The mean particle size of all formulations was ranged between 162.2 ± 2 nm to 191.0 ± 8 nm and PDIs value was ranged between 0.341 to 0.498. Above all formulation, EF5 exhibited the maximum encapsulation efficiency with mean particle size of 162.2 ± 2 nm and a PDI of 0.341. Vesicle size play an important role in topical drug delivery systems as small size vesicles delivers their contents more efficiently across deeper layers of skin [20, 21]. In our investigation formulated ethosomes exhibited average diameter of 191.0 ± 8 nm and confirmed their suitability for drug delivery across skin. In the present study, it was observed that on increasing the ethanol and soya lecithin concentration above 35% and 200 mg respectively, particle size was increased drastically and showed narrow distribution for polydispersity index. Therefore either increase or decrease in the ratio of lipid and ethanol widely affect the physical properties of ethosomes. Particle size and ethanol concentration influences the skin permeability as only small sized particles can penetrate through the skin and ethanol concentration remarkably influences drug permeability [22]. Considering the above results it can be concluded that particle size of EF5 was best suited for topical application [23, 24].

**Table 4:** Characterization of flurbiprofen loaded ethosomes

Characterization	EF1	EF2	EF3	EF4	EF5	EF6	EF7	EF8	EF9
Vesicle Size (d.nm)	183.0 ± 3	179.2 ± 6	177.8 ± 5	173.8 ± 6	162.2 ± 2	191.0 ± 8	181. ± 9	190. ± 2	183.5 ± 1
% Entrapment efficiency	82 ± 2	85 ± 1%	83.5 ± 1	87 ± 1	92.1 ± 1	86.2 ± 2	88.2 ± 2	82.9 ± 2	90 ± 1
PDI	0.369	0.418	0.371	0.398	0.341	0.498	0.357	0.389	0.344
Zeta Potential (mV)	−29.4 ± 0.5	−43.4 ± 1.8	−27.3 ± 2.3	−31 ± 2.4	−48.14 ± 1.4	−38.7 ± 3.1	− 34 ± 0.2	−29.3 ± 1.3	−35.1 ± 0.2
J (flux) (μg/cm2/h)	152.2	140.4	190.5	150.45	226.1	201.4	169.8	194.3	164.1
pH	6.6	5.4	6.1	6.3	5.2	5.4	6.4	5.1	5.1
Viscosity (cps)	7104 ± 0.6	6342 ± 1.4	7534 ± 2.43	7132 ± 3.12	8539 ± 1.1	7432 ± 1.3	6321 ± 1.8	7322 ± 2.1	6076 ± 1.2
Spreadability (cm)	4.1 ± 3.1	6.84 ± 0.3	7.22 ± 2.3	6.4 ± 0.5	7.12 ± 0.3	6.43 ± 0.5	6.54 ± 3.2	4.3 ± 3	3.2 ± 0.3

The stability of ethosomal formulation was carried out by measuring zeta potential. The zeta potential of different ethosomal formulations from EF1 to EF9 showed value ranging from − 27.3 ± 2.3 mV to − 48.14 ± 1.4 mV (Table 4). Formulation EF3 showed minimum (− 27.3 ± 2.3 mV) zeta potential while EF5 showed the maximum (− 48.14 ± 1.4 mV) value. EF1, EF4 and EF8 exhibited no significant change showing values − 29.4 ± 0.5, − 31 ± 2.4 and − 29.3 ± 1.3, respectively. EF2, EF6 and EF9 showed increased zeta potential value i.e. -43.4 ± 1.8, − 38.7 ± 3.1 and 35.1 ± 0.2 respectively. Above findings indicated that, the particles in the suspension carried anionic charges and an increase in soya lecithin concentration, further increased anionic charges in the system, and thus resisted agglomeration [17]. Therefore, increase in the surface charge resulted in augmentation of ethosomes stability. From the above results ethosomal formulations EF5, showed maximum stability prepared by thin film hydration method. The results obtained from the zeta potential measurement study, gives us the idea that the charge carried by the vesicles get influenced due to the variation in vesicle sizes.

#### Morphology of Flurbiprofen loaded ethosomes by TEM, SEM, and AFM

**Fig. 1:**
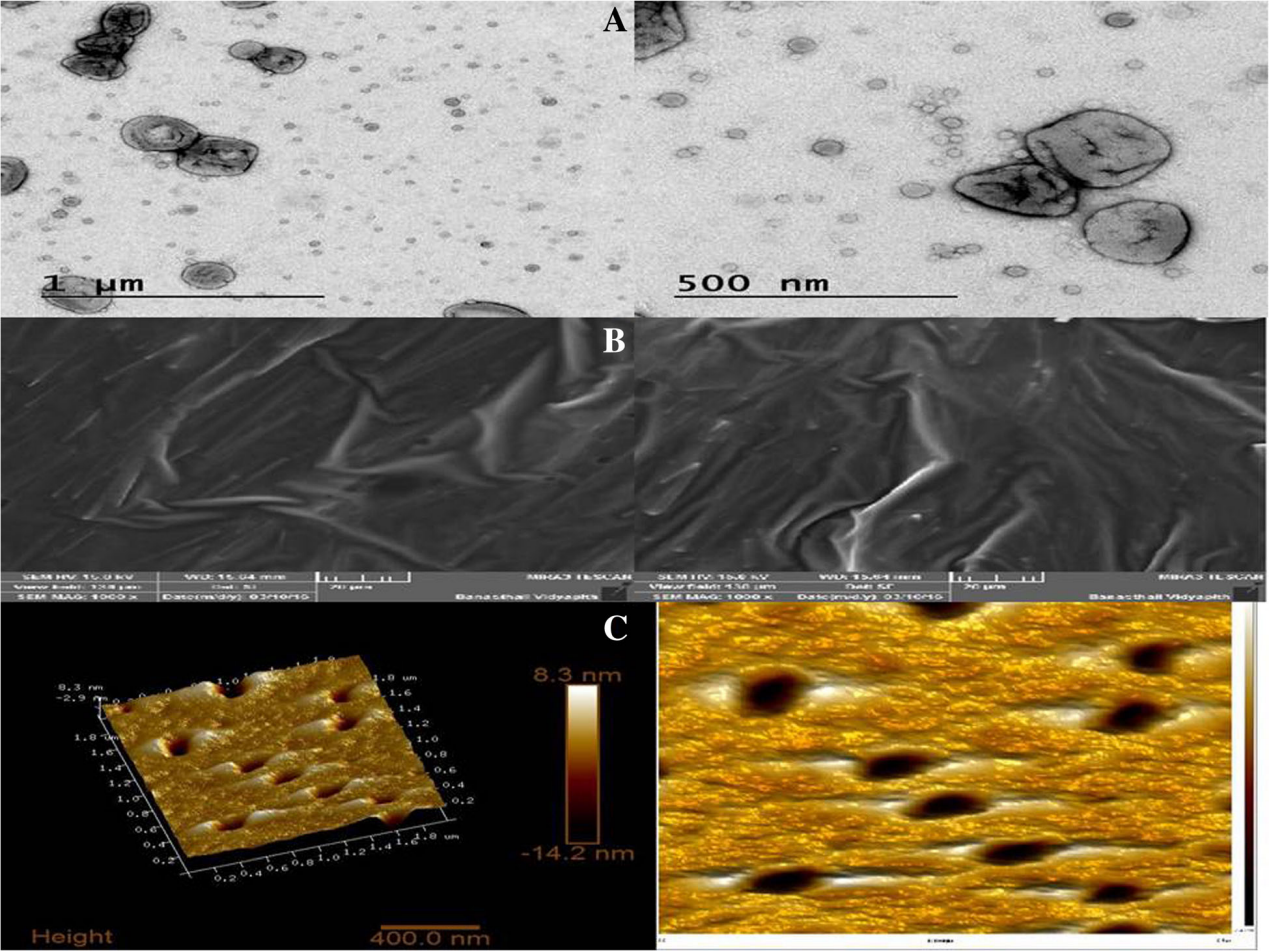
Surface micrograph of optimized formulation EF 5 shown by [**a**] TEM [**b**] SEM [**c**] AFM

The external morphology of the prepared optimized formulation EF5 is shown in Fig. 1a. The formulated ethosomes were well acknowledged with their predominantly spherical texture. The images captured suggested that ethosomes formed were round, smooth, free from drug crystalline structures and multilamellar in nature. The dense particle in the SEM image suggested that lipid was of high density and helped in the controlled release of drug. SEM image of flurbiprofen loaded ethosomal formulation EF5 was shown in Fig. 1b indicated that ethosomes were spherical in shape with clear visible defined boundaries with the nano size range. TEM further helps in confirmation of surface morphology of the optimized formulation, and its finding clearly demonstrated that ethosomes were round, smooth and free from drug crystals.

Further analysis of formulation with AFM images exposed information’s regarding behavior of the carriers that cannot be gained so easily by the SEM and TEM images. AFM images of ethosomal formulation were shown in Fig. 1c that provides various facts about the morphological characteristics of prepared ethosomal formulation. Furthermore, the external morphology validated the height and diameter of the molecules. Height of the ethosomes were found as 1.712 nm, area 4138.570 nm^2^, diameter 59.899 nm and density 14.597(/μm^2^). These results noticeably predicted that the interaction with the substrate resulted in an even smooth spherical shape since the measured diameter of the average sized structure was 179 nm. No cracks or pinholes were visualized in ethosomes. Ethosomal formulations size were in the range of 162–191 nm [25, 26].

#### Attenuated total reflection Fourier-transform infra-red spectroscopy

**Fig. 2:**
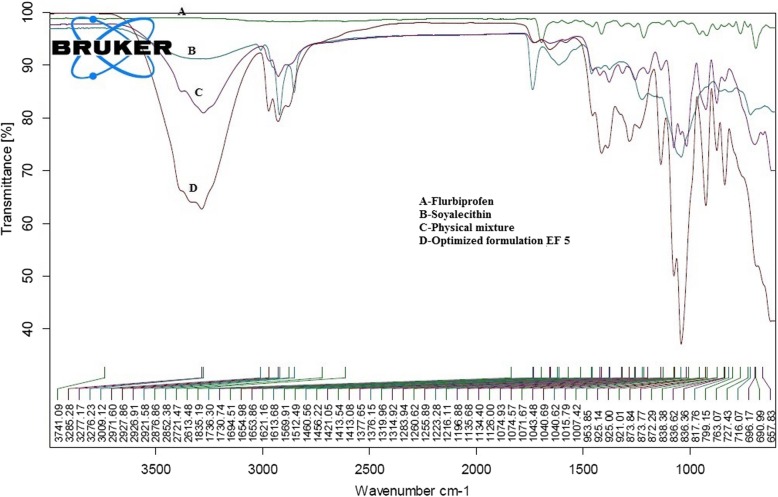
Overlapped ATR-FTIR data [**a**] Flurbiprofen [**b**] Soya lecithin [**c**] Physical mixture [**d**] Optimized formulation EF 5

The ATR-FTIR results of pure drug, soya lecithin and flurbiprofen loaded ethosomal formulation (EF5) was depicted in Fig. 2. ATR-FTIR spectra of the drug showed major characteristic peaks at 3741.09 cm^− 1^ due to 0-H stretching, 2921.58 cm^− 1^ due to = CH and aromatic H stretching, and 2852.38 cm^− 1^ due to CH_2_ stretch, 1731.74 cm^− 1^ due to C=O stretching, 1261.16 cm^− 1^ due to O–H bending, 1040.62 and 1070.93 cm^− 1^ due to C–F bending and 836.36 cm^− 1^ due to distribution of aromatic protons. The above results showed that there was no considerable alteration in the IR peaks of flurbiprofen and optimized formulation specifying the absence of any interactions between the drug and polymer. Further, the above results were also validated by DSC analysis.

#### Raman spectroscopy

**Fig. 3:**
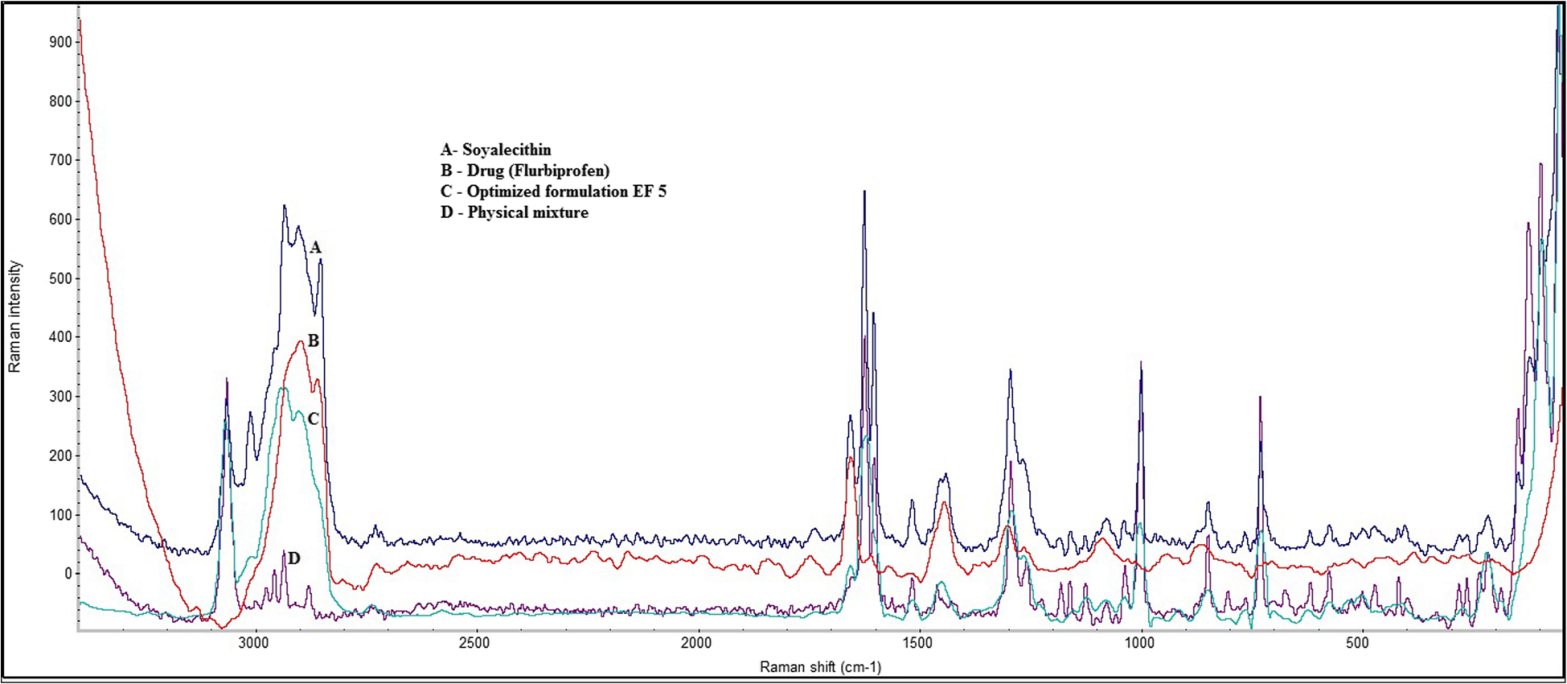
Overlapped RAMAN spectra [**a**] Soyalecithin [**b**] Drug (Flurbiprofen) [**c**] Optimized formulation EF 5 [**d**] Physical mixture

Raman spectroscopy is dependent on the electric polarizability of the molecule and gives an idea about the functional group present in the sample. The major peak of drug (Flurbiprofen) were shown at 3063 cm-^1^ (−OH stretching), 3071 cm-^1^ (aromatic CH_2_ stretching), 1615 cm ^− 1^ (−OH bending), 1300 cm-^1^ (CH_3_ bending), 1033 cm-^1^ (C-F) while formulation EF-5 showed peak at 3010 cm-^1^, 2904 cm-^1^, 2856 cm-^1^, 1658 cm-^1^, 1445 cm-^1^, 1303 cm-^1^, 1003 cm-^1^, 1089 cm-^1^. The soyalecithin peaks were showed at 2853 cm^− 1^, 2193 cm^− 1^, 2030 cm^− 1^, 1825 cm^− 1^, 1730 cm^− 1^, 1653 cm^− 1^, 1300 cm^− 1^and physical mixture showed peaks at 2857 cm^− 1^, 2726 cm^− 1^, 1732 cm^− 1^, 1442 cm^− 1^, 1299 cm^− 1^, 1076 cm^− 1^. All the components present in the formulation along with its physical mixture were scanned and it was observed that there were least interaction between the different samples and peak of optimized formulation were overlapping with drug which show the presence of drug in ethosomal formulation. Data was matched with the FTIR for the presence of different functional group. Overlapping peaks was represented in Fig. 3.

#### DSC analysis

**Fig. 4:**
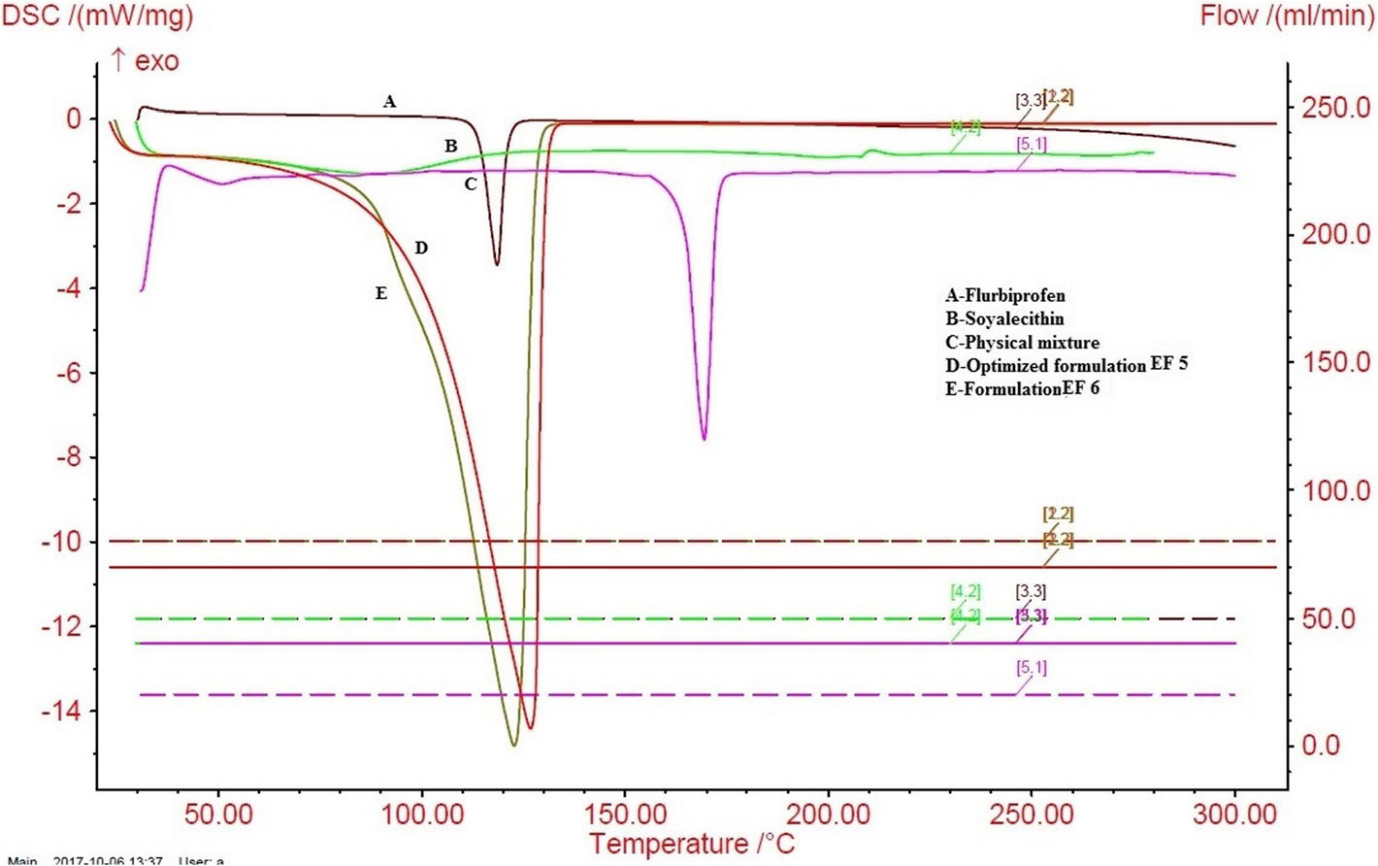
Overlapped DSC Thermogram [**a**] Flurbiprofen [**b**] Soya lecithin [**c**] Physical formulation [**d**] Optimized Formulation ES 5 [**e**] Formulation EF 6

With the help of DSC instrument, thermal behavior and various interactions between drug (flurbiprofen), lipid (Soya lecithin) and physical mixture were studied. Results of DSC experiments showing endothermic peaks of drug, drug loaded ethosomes and physical mixtures (drug + lecithin) were depicted in Fig. 4. The DSC thermogram of flurbiprofen exhibited a sharp and high intensity peak at 117 °C. Broad endothermic and low intensity soya lecithin peak was found at 87 °C. The melting point for optimized formulation EF5 showed sharp and high endothermic peak at 121 °C which was found very close to our standard drug flurbiprofen. The peak of formulation EF1was found at 129 °C. The melting point for optimized formulation showed peak of high intensity compared to the pure drug. On increasing ethanol concentration from 30 to 35% the phase transition temperature decreases. With the addition of ethanol up to 40%, the phase transition behaviour disappeared. However the transition enthalpy and entropy get increased with the increase in ethanol concentration [27]. This implies that our optimized formulation EF5 shows no interaction with other excipients. DSC helps in critical evaluation of the effectiveness and absolute purity determination of drugs. The result shows good compromise between accuracy, resolution and sensitivity.

#### Thermo gravimetric analysis (TGA)

**Fig. 5:**
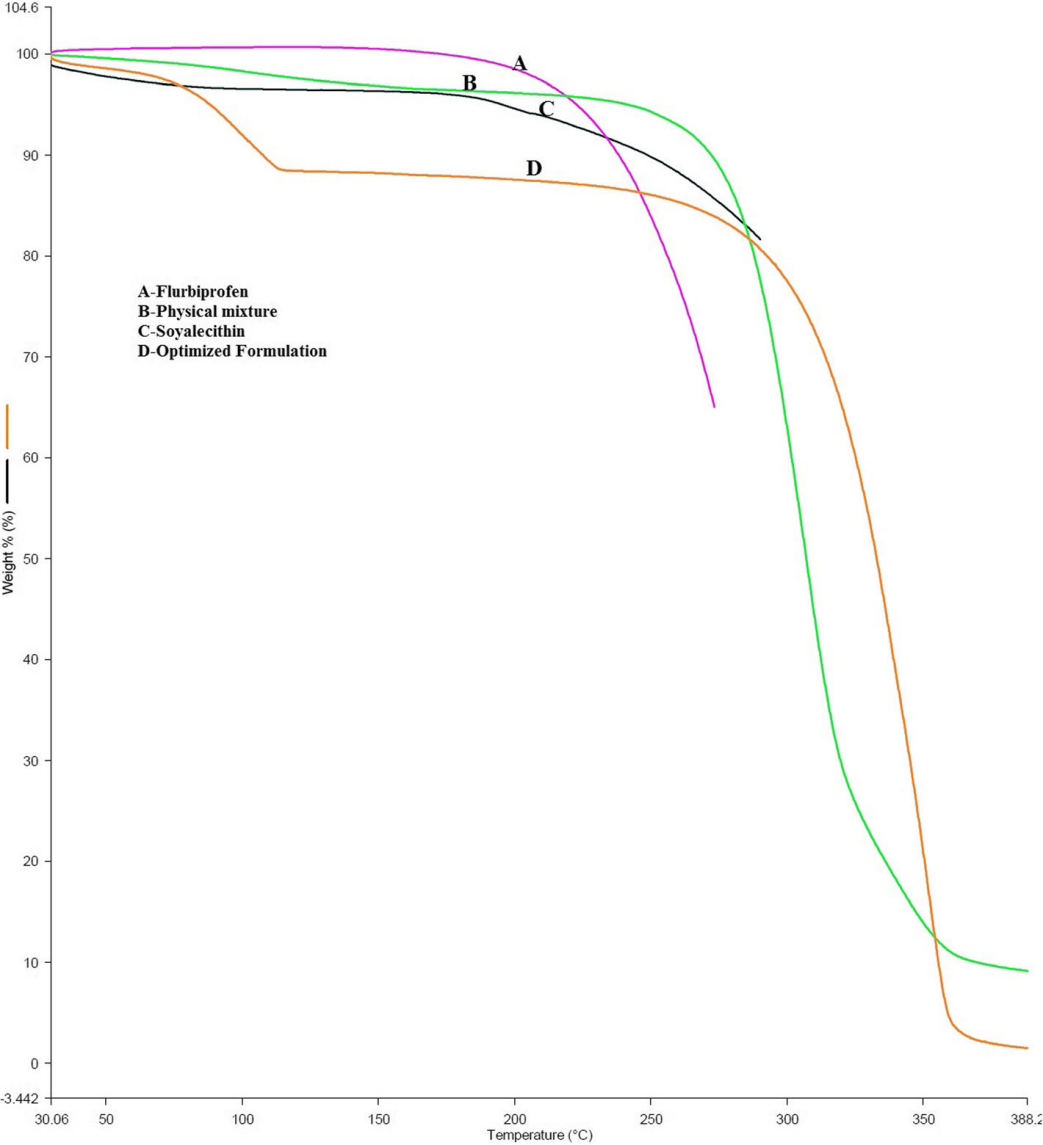
Overlapped TGA thermograph: [**a**] Flurbiprofen [**b**] Physical Mixture [**c**] Soya lecithin [**d**] Optimized formulation EF 5

In the present study, TGA was performed to measure the change in mass of the sample with respect to temperature change. The experiment involves the study of flurbiprofen, soya lecithin, physical mixture and optimized formulation (EF5) in order to examine their thermal property. The % weight loss and the melting temperature for the samples are depicted in Figure as A) Flurbiprofen B) Physical mixture C) Soya lecithin and D) Optimized formulation EF5. The TGA weight loss % for flurbiprofen, Soya lecithin and physical mixture was found to be 97, 94 and 92% at 200 to 250 °C, 250 to 300 °C and 250 to 275 °C respectively. Optimized formulation EF5 showed instant 88% weight loss at 100 to 120 °C and became constant till 300 °C. From the above TGA result, it is clearly indicated that the mixture of soya lecithin with the drug improves its stability. TGA shows a useful means for evaluating the weight loss of the polymers and drug. All these outcomes discovered that excipients or moisture content have no or minimal opposing effects on the formulations. Overlapped TGA spectra was shown in Fig. 5.

#### Entrapment efficiency of ethosomes

**Fig. 6:**
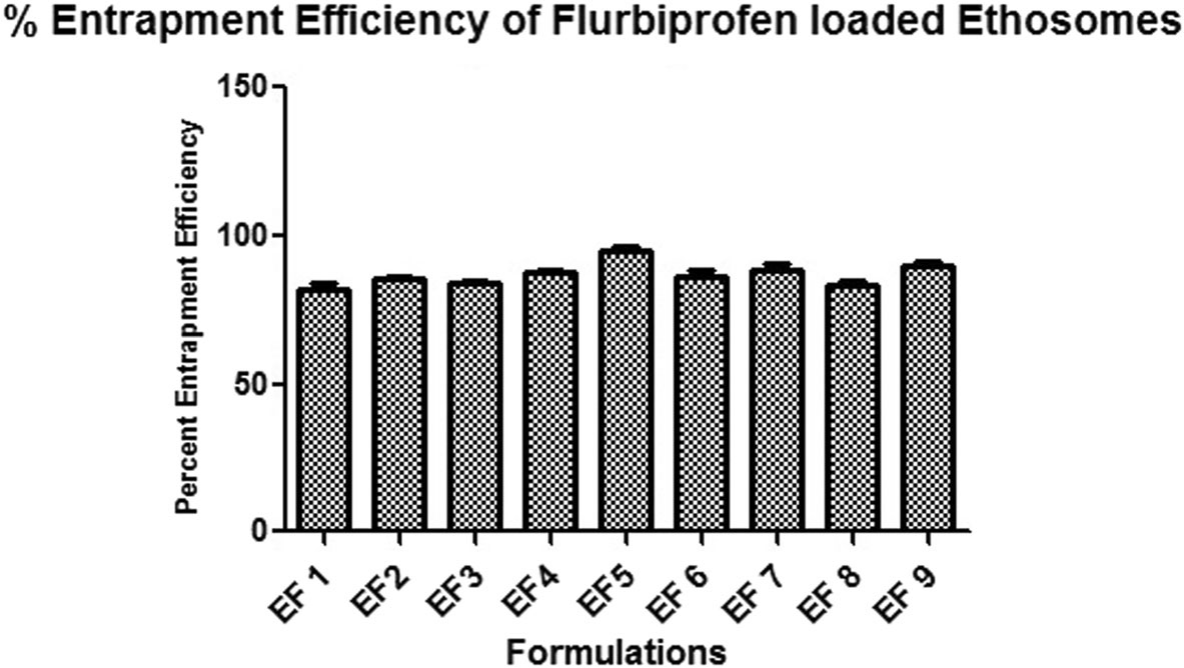
Percent entrapment efficiency of formulation EF 1-EF 9

Entrapment efficiency is an important parameter that assesses the delivery potentiality of a system. The entrapment efficiency of all formulations with different concentration of flurbiprofen loaded ethosomes were determined and summarized in Table 4. In the midst of all the formulations, EF5 was found to have maximum 92.1 ± 0.1% and EF1 showed the minimum 82 ± 2% encapsulation efficiency. Maximum entrapment efficiency of optimized formulation EF5 was contributed due to the presence of average amount of lipid and ethanol i.e. 200 mg 35% respectively. Formulation EF1, EF2, EF4 and EF6 showed no significant difference in entrapment efficiency showing values 82 ± 2%, 85 ± 1%, 87 ± 1% and 86.2 ± 2% respectively. However, decreased value of entrapment efficiency was observed in EF3, EF8 and EF9 compared to rest formulations [17, 28]. The entrapment efficiency was increased on increasing alcohol content up to 35%, however efficiency decreased when the ethanol concentration was increased above 35%. This could be due to the higher permeation enhancing property of ethanol that leads to the leakage of lipid bilayer. Also, the entrapment efficiency increases with increase in concentration of lecithin up to 200 mg but, above 200 mg of lecithin concentration drug permeability was reduced. However, it was also observed that formulation EF6 containing same amount of lipid i.e. 200 mg showed less entrapment efficiency, this could be due to the increased ethanol concentration i.e. 40% in the formulation. Also, it is commonly known that high concentration of ethanol cannot co-exist with lipid vesicles. From the above results, it can be concluded that particle size and entrapment efficiency of ethosomes formulation could be significantly affected by varying the ethanol and lipid concentration and by keeping other variables constant [29]. The order for entrapment efficiency was found to be EF5 > EF9 > EF7 > EF4 > EF6 > EF2 > EF3 > EF8 > EF1 and it was graphically shown in Fig. 6.

#### Viscosity, pH and Spreadability

The viscosity, pH and spreadability of the gel were found to be 6076 ± 1.2 to 7534 ± 1.2, 5.1 to 6.6 and 32 ± 0.3 to 7.22 ± 0.3 cm respectively which are suitable for the application over skin and the results were shown in Table 4. Above optimized formulation EF5 displayed sufficient viscosity, pH and spreadability which revealed a good gelling property of ethosomal gel for topical application. Results of the study revealed that, ethanol and lipid concentration for formulation EF5 was found to be optimized considering the all parameters mentioned in Table 4 [28, 30]. Further ethosomal suspension was incorporated into 1% Carbopol gel for topical application. Ethosomal formulation of apigenin having anti-inflammatory, anti-oxidant and anti-carcinogenic properties showed enhanced skin deposition and transdermal flux with increase in the level of phospholipid and ethanol concentration [31]. High ethanol concentration plays major role in the formulation of ethosomes [32].

#### Skin irritation assay in rat

The skin irritation assay was performed by applying free drug and optimized formulation EF5 on rat skin and observed for 72 h. The moderate to severe erythema was observed in terms of Primary irritation index (PII) for both the test compounds i.e. drug and EF5. For free flurbiprofen solution PII was found as 1.6 which restricts the suitability and tolerability by the recipients. In contrary, EF5 formulation caused less irritation with PII of 1.0. Flurbiprofen is commensally associated with rashes and rarely with encrusted skin. So it cannot be used directly by the patients. Flurbiprofen loaded ethosomes should be able to reduce the irritation due to the increased encapsulation efficiency of the drug. Therefore, EF5 can be reliably used in dermal issues and can be considered safe for topical formulation. Flurbiprofen is associated with noticeable skin problems like skin rashes, which strongly restricts their applicability and acceptability by the patients. Ideally, the flurbiprofen containing ethosomes could be able to reduce irritation and improves patient acceptability.

#### In vitro skin permeation study

**Fig. 7:**
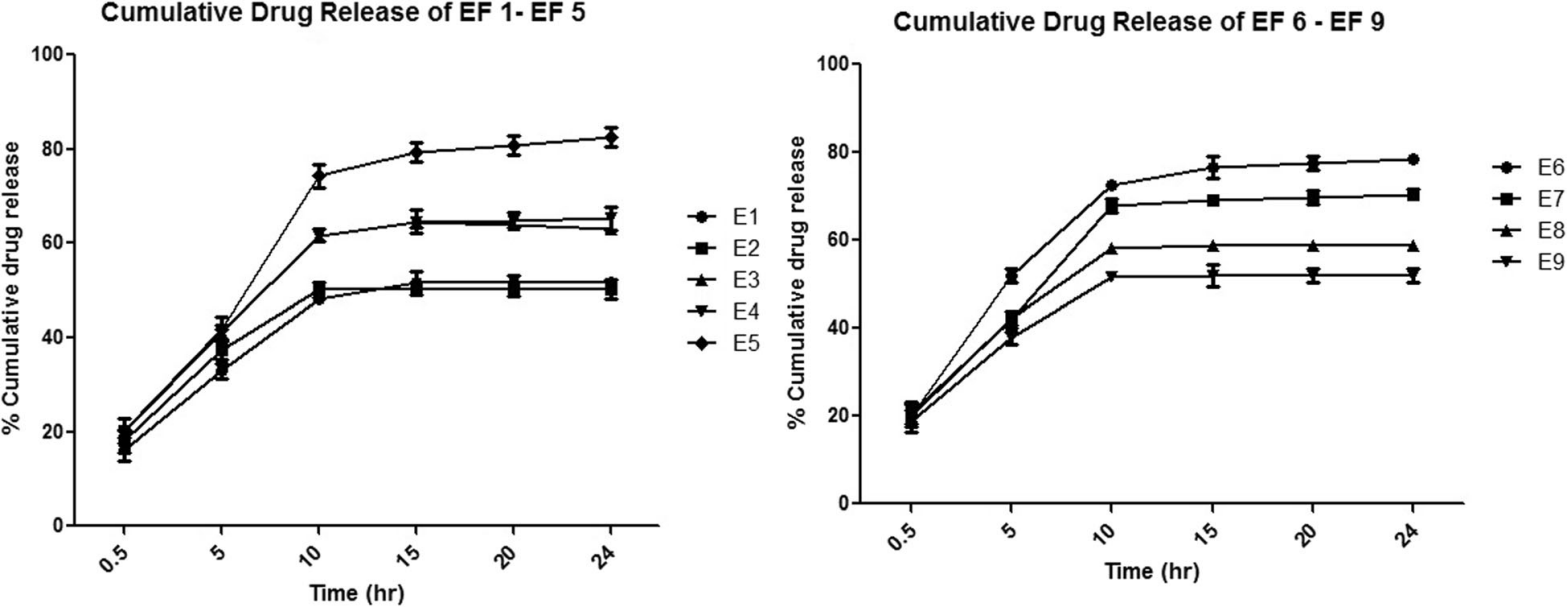
In-vitro skin permeation study profile of flurbiprofen loaded ethosomal formulations EF 1- EF 9 for 24 h

The In vitro skin permeation studies for various formulations were studied using Albino rat skin. The change in ethanol and lipid concentrations plays an important role in the release of flurbiprofen from ethosomal formulation. The results of transdermal skin permeation of different formulations from EF1 to EF9 were shown in Fig. 7. The drug permeation study through rat skin was examined for 24 h and the formulations EF1 to EF9 showed cumulative results as 51.59 ± 1.12, 50.12 ± 2.01, 63.16 ± 1.01, 63.23 ± 2.52, 82.56 ± 2.11, 78.52 ± 1.21, 70.25 ± 1.21, 58.75 ± 1.01 and 51.85 ± 1.52 g/cm^2^ respectively. From the above results it was clear that EF5 showed maximum release i.e. 82.56 ± 2.11 g/cm^2^, possibly due to the greater entrapment of drug in ethosomes in comparison to other formulations. Initially ethosomes shows burst release of drug and then sustained release thereafter. The sustained release is accredited to the presence of lipid bilayer composed of lipid that acts as a rate limiting membrane for release of the encapsulated drug [32]. A rapid release was obtained for EF1 and EF2 formulations which had low lipid concentration. Also, there was no significant difference in drug permeation for the formulation EF7, EF8 and EF9 prepared with the high concentration of lipid 300 mg and varied concentration of ethanol. EF2 showed minimum amount of drug permeation 50.12 ± 2.01 g/cm^2^ due to less encapsulation efficiency. The obtained optimized formulation EF5 followed Korsmeyer-peppas release pattern and anomalous (non-Fickian) transport behavior. From the above results it can be predicted that variation in lipid and ethanol concentrations may modify the drug release patterns in all formulations [23].

#### Data treatment result

**Fig. 8:**
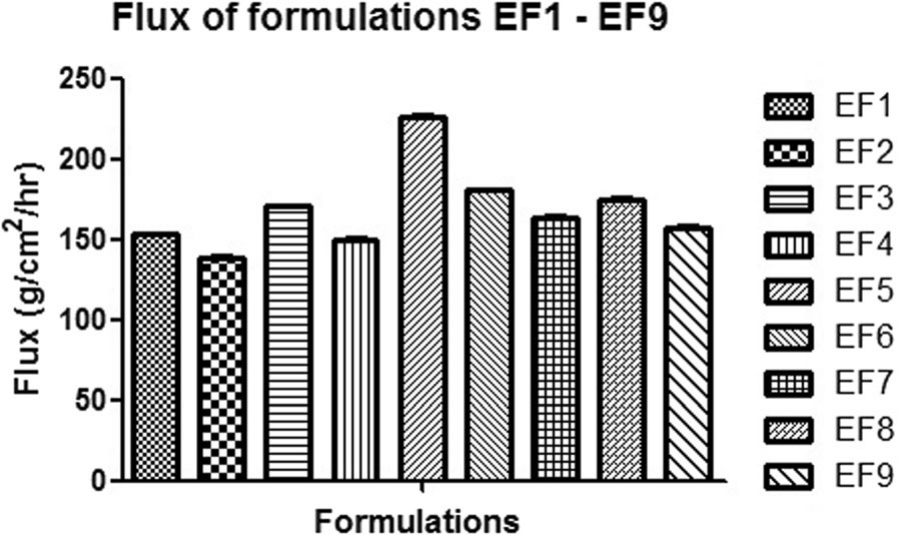
Flux of the formulation EF 1- EF 9

The data obtained from in vitro skin permeation study were further treated mathematically to obtain the value of flux for all formulations. The flux value for all formulations from EF1 to EF9 lies between 140.4 to 226.1 μg/cm^2^/h. Optimized formulation EF5 showed maximum flux value 226.1 μg/cm^2^/h. The results were tabulated in Table 4 as well as depicted in Fig. 8. Flurbiprofen loaded ethosomes provided better flux as compared to the other reported formulations and also provided a greater skin deposition, thus qualifying its use as a carrier for choice in dermal and transdermal delivery. Ethosomal formulation has been reported as non-irritant and well tolerated in vivo [33, 34]. Differences in the flux values were indicative of varying concentration of lipid: ethanol ratio in formulations.

#### Cold plate test

**Fig. 9:**
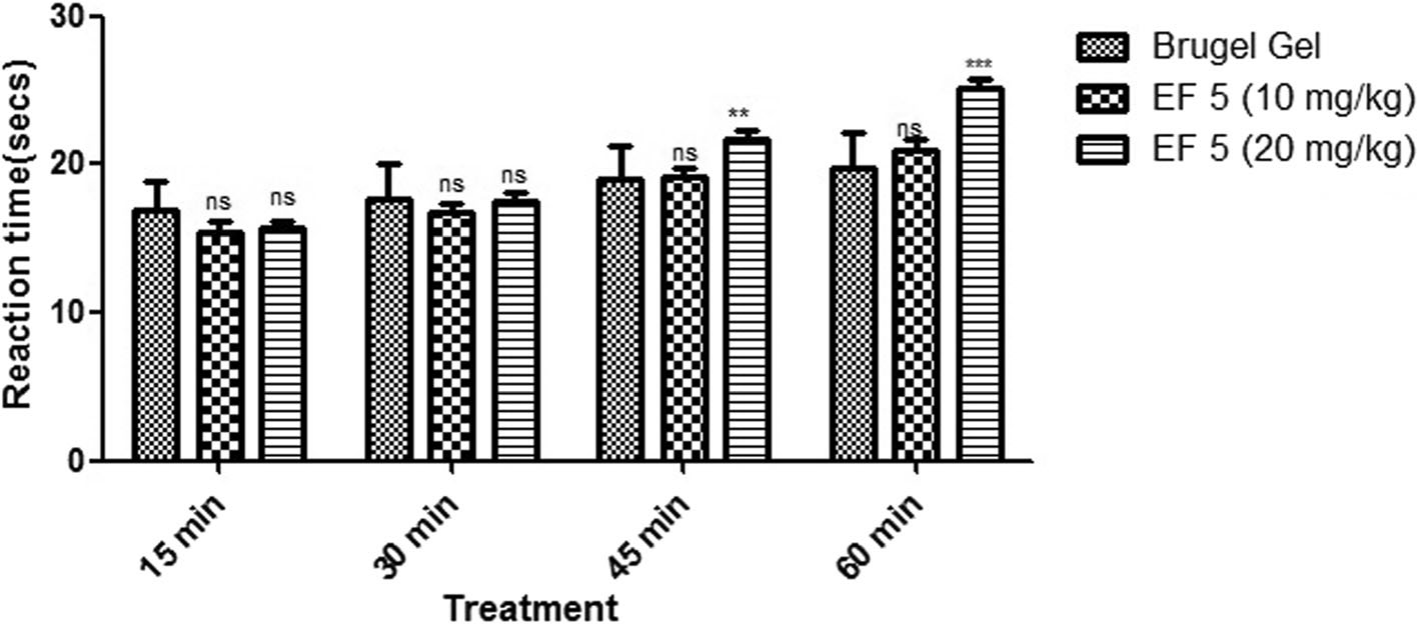
Effect of EF 5 formulations on reaction time in cold plate test

The formulations showed dose dependent increase in the reaction time for 10 and 20 mg/kg as similar to Brugel® marketed preparation markedly (Table 5, Fig. 9). EF 5 formulations at dose of 10 mg/kg (from 15.33 ± 0.51to 21 ± 0.63 min) and 20 mg/kg (15.66 ± 0.81 to 25.16 ± 0.40 min) caused remarkable increase in reaction time (Additional file 1). The cold plate test has been considered as an ideal test for evaluation of centrally acting therapeutic agents. Formulations EF 5 showed marked analgesic activity, which could be due to involvement of opioid receptors. The opioid agents exert their analgesic action via the supraspinal (μ1, k3, δ1, σ2) and spinal (μ2, k1, δ2) receptors. From our result, it can be concluded that it is possible that our formulations exert its effect through the central opioid receptor or promoted release of endogenous opioid peptides [35]. R-flurbiprofen demonstrates potent analgesic activity via inhibition of COX and possess minimal toxicities.

**Table 5:** Screening of analgesic activity of selected formulations using cold plate test in rats

S.No.	Treatment	Reaction time (sec.)			
		15 min	30 min	45 min	60 min
I	Brugel Gel	16.9 ± 2.02	17.7 ± 2.4	19 ± 2.21	19.8 ± 2.41
II	Etho 5 (10 mg/kg)	15.33 ± 0.51^ns^	16.8 ± 0.63^ns^	19.2 ± 0.63^ns^	21 ± 0.63^ns^
III	Etho 5 (20 mg/kg)	15.66 ± 0.81^ns^	17.48 ± 0.5 ^ns^	21.66 ± 0.51^**^	25.16 ± 0.40^***^

*ns* Not significant

**Significant

***Highly significant

#### Carrageenan induced paw edema

**Fig. 10:**
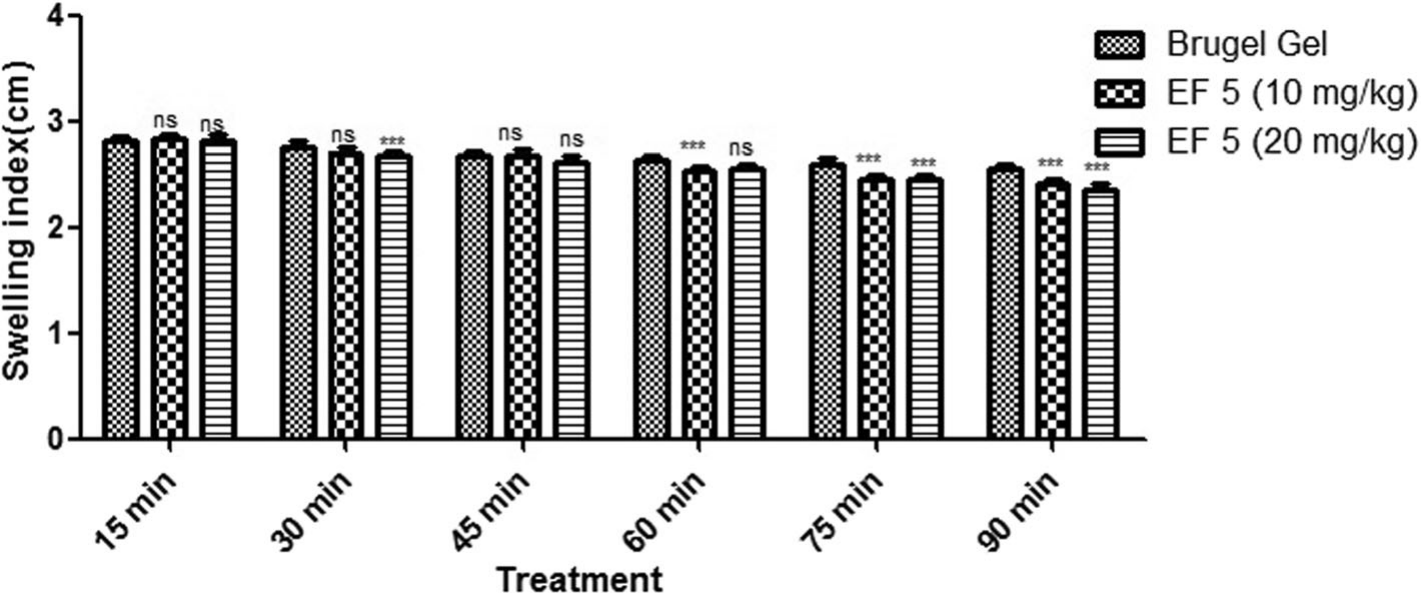
Effect of formulations on swelling index in carrageenan induced inflammation

As shown in Table 6, Fig. 10, EF 5 formulation at 10 mg/kg inhibited the increase in paw edema (2.83 ± 0.04 to 2.41 ± 0.05 min) from 15 min to 90 min. Similarly, EF 5 at 20 mg/kg significantly inhibited paw edema (2.82 ± 0.06 to 2.35 ± 0.05) from 15 min to 90 min as compared to Brugel® marketed preparation. A dose dependent inhibition in paw edema was observed for both the formulations at 10 and 20 mg/kg. Carrageenan induced paw edema is a widely used model to assess anti-inflammatory capacity of any compound. A biphasic response is usually achieved after parentarally administered carrageenan, i. e. first phase of 15–45 min includes release of mediators like bradykinin, serotonin and histamine while second phase of 60–90 min implicates release of prostaglandins only. Carrageenan-induced rat paw edema model was used to investigate anti-inflammatory activity of flurbiprofen proniosomes [36]. In vivo interaction studies of ACE inhibitor (Lisinopril) with commonly used NSAIDs flurbiprofen and ibuprofen in carrageenan induced inflammation (CII) rats to check the anti-inflammatory response of NSAIDs with Lisinopril and alone [37].

**Table 6:** Screening of anti-inflammatory activity of selected formulation in carrageenan induced paw edema in rats

S.No.	Treatment	Swelling index (cm)					
		15 min	30 min	45 min	60 min	75 min	90 min
I	Brugel Gel	2.81 ± 0.04	2.76 ± 0.05	2.68 ± 0.04	2.63 ± 0.05	2.59 ± 0.06	2.55 ± 0.05
II	Etho5 (10 mg/kg)	2.83 ± 0.05^ns^	2.7 ± 0.06^ns^	2.66 ± 0.05^ns^	2.53 ± 0.05^***^	2.45 ± 0.05^***^	2.41 ± 0.04^***^
III	Etho5 (20 mg/kg)	2.82 ± 0.06^ns^	2.67 ± 0.05^***^	2.62 ± 0.06^ns^	2.55 ± 0.05^ns^	2.45 ± 0.05^***^	2.35 ± 0.05^***^

*ns* Not significant

**Significant

***Highly significant

## Conclusion

The development of a suitable carrier for dermal administration of flurbiprofen has been highly challenging due to its hydrophilic nature, resulting in limited liberation into the skin. The formation of more lipophilic carrier for flurbiprofen for adequate solublization capacity and enhanced penetrative properties would be expected to increase the bioavailability of the drug. Ethosomes appears to be the suitable carrier for flurbiprofen for dermal delivery by entrapping the drug in between the lipophilic layer. Several reports have advocated that an ethosomal formulation provides consistent skin permeability and enhanced drug permeation from lipid vesicles having ethanol as one of key components. Therefore the goal of present study was to develop ethosomal delivery of flurbiprofen. Notably, our current investigation revealed that flurbiprofen loaded ethosomes as potentially useful vehicle. They have provided ideal particle size, PDI, enhanced transdermal flux, higher encapsulation efficiency with lower skin irritancy and higher stability at 4 °C/60 ± 5 RH. Delivery of drug by ethosomes was significantly influenced by variations in lipid and ethanol concentrations in formulations. Thus, the overall study concluded that this ethosomal approach offers a new delivery system for sustained and targeted delivery for flurbiprofen. Based on this approach, future research works is needed to bring the flurbiprofen loaded ethosomal formulation closer to its clinical realization.

## Additional file

Additional file 1: Raw data for anti-inflammatory actvity is available in additional file. Raw observations for preclinical study is available in supplementary file. (DOC 1135 kb)

## Abbreviations

%: Percentage
API: Active pharmaceutical ingredient
CDR: Cumulative drug release
cm: Centimeters
DSC: Differential scanning calorimetry
FDA: Food and Drugs Administration
FTIR: Fourier transformed infra red
g/L: Gram per litre
gm/ml: Gram per millilitre
h: Hour
ICH: International Conference on Harmonization
J: Flux
NSAID: Non-steroidal anti-inflammatory drug
PDI: poly dispersive index
PL: Phospholipids
rpm: Rotations per minute
SC: Stratum corneum
SEM: Scanning electron microscopy
TDDS: Transdermal drug delivery system
TEA: Triethyl amine
UV: Ultra violet
μ: Microns
