# Supplementary material for: Flurbiprofen loaded ethosomes - transdermal delivery of anti-inflammatory effect in rat model
Source: Lipids Health Dis. 2019 Jun 7;18:133. doi: 10.1186/s12944-019-1064-x (PMC6554971; doi:10.1186/s12944-019-1064-x)
Supplement: Supplementary file 1 — Raw data for anti-inflammatory actvity is available in additional file. Raw observations for preclinical study is available in supplementary file. (DOC 1135 kb) [file 12944_2019_1064_MOESM1_ESM.doc]

**SUPPLEMENTRAY DATA**

**IR spectra**

**IR of Flurbiprofen**


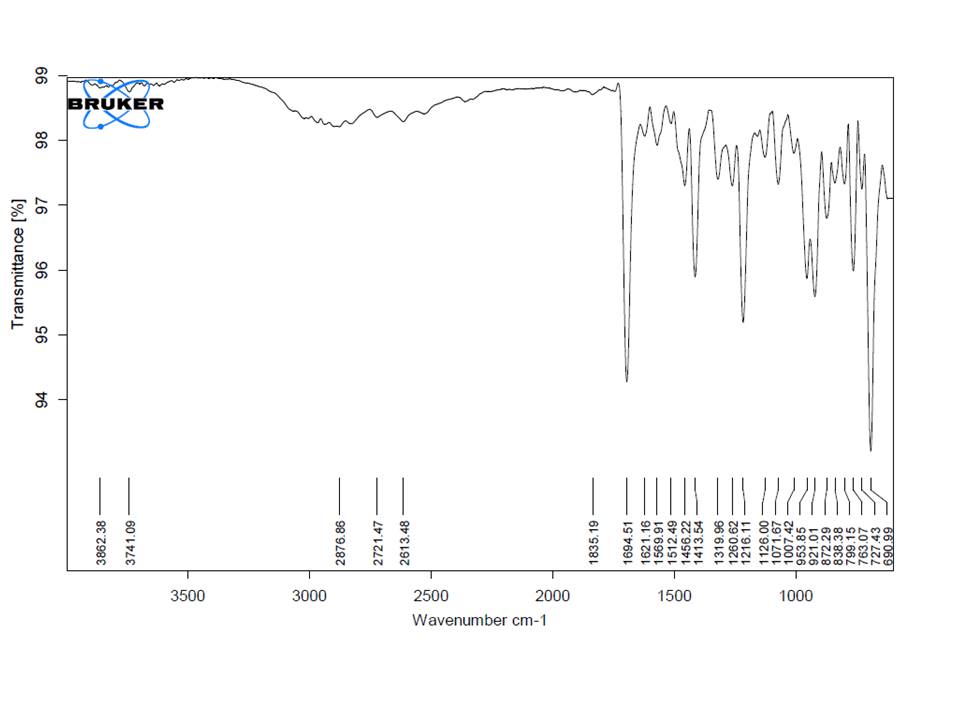


**IR of soyalecithin**


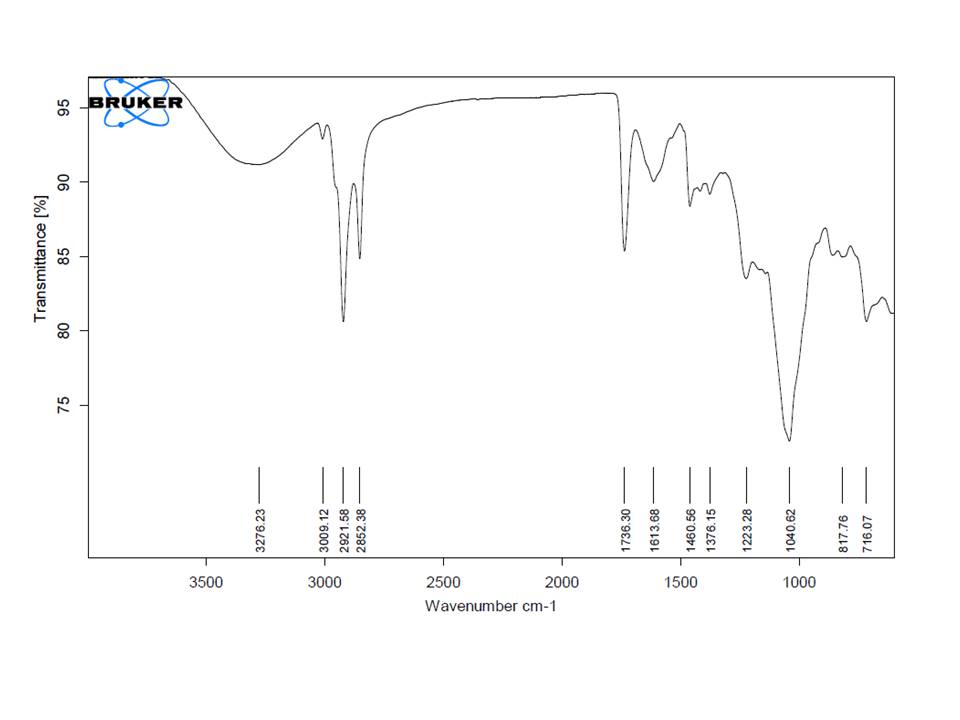


**IR of optimized formulation**

**
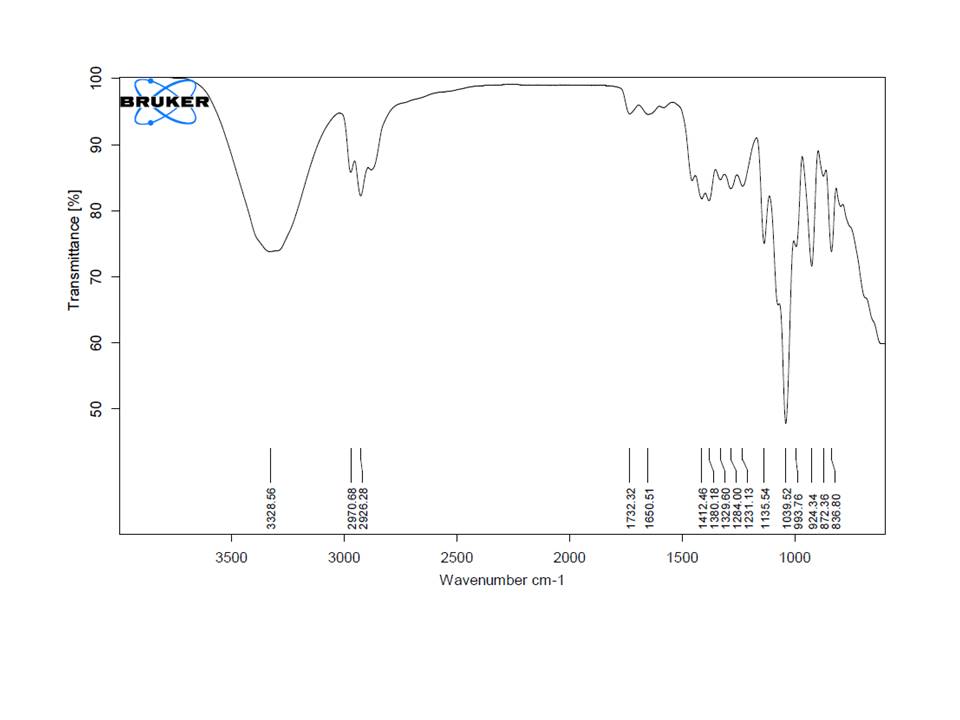
**

**RAMAN spectra**

**RAMAN spectra of flurbiprofen**

**
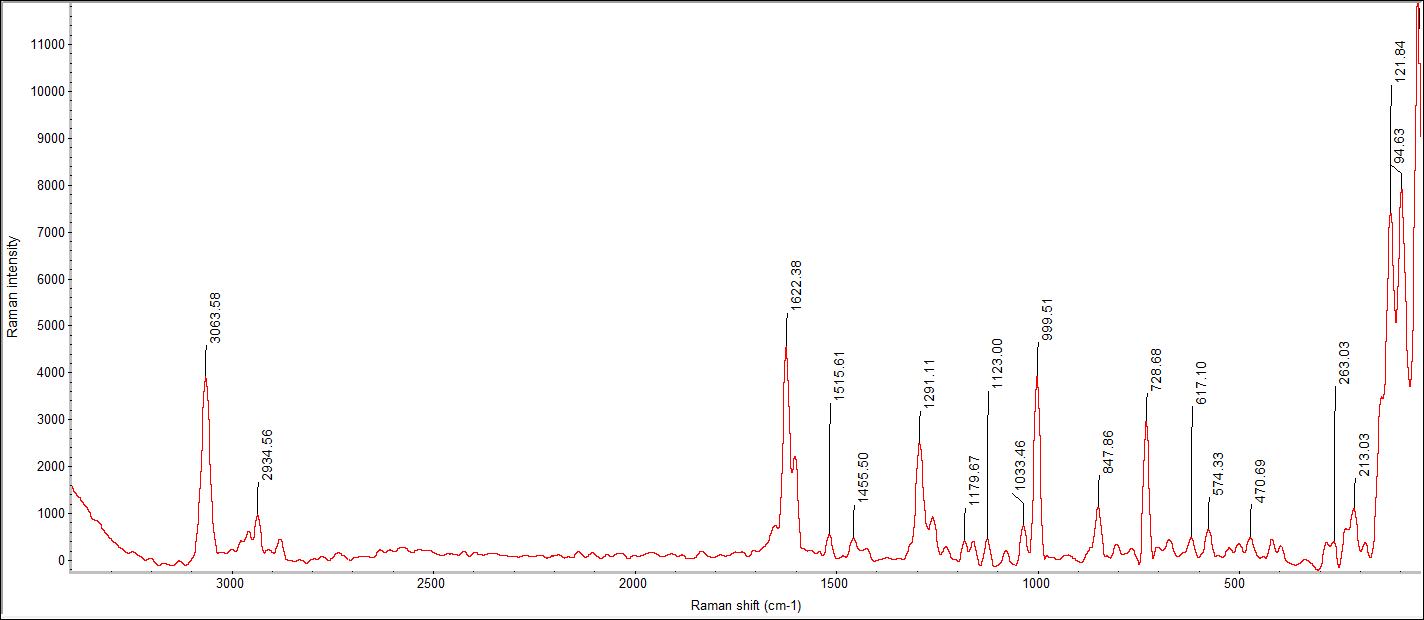
**

**RAMAN spectra of soyalecithin**

**
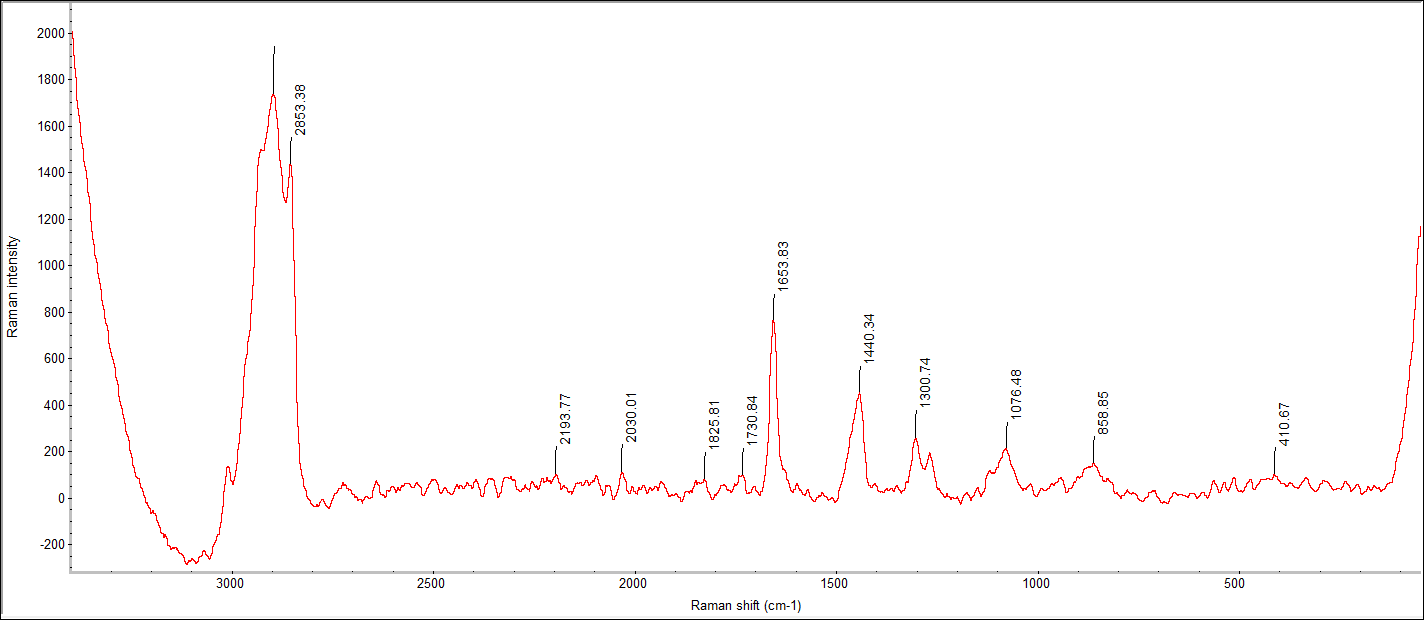
**

**RAMAN spectra of optimized formulation**

**
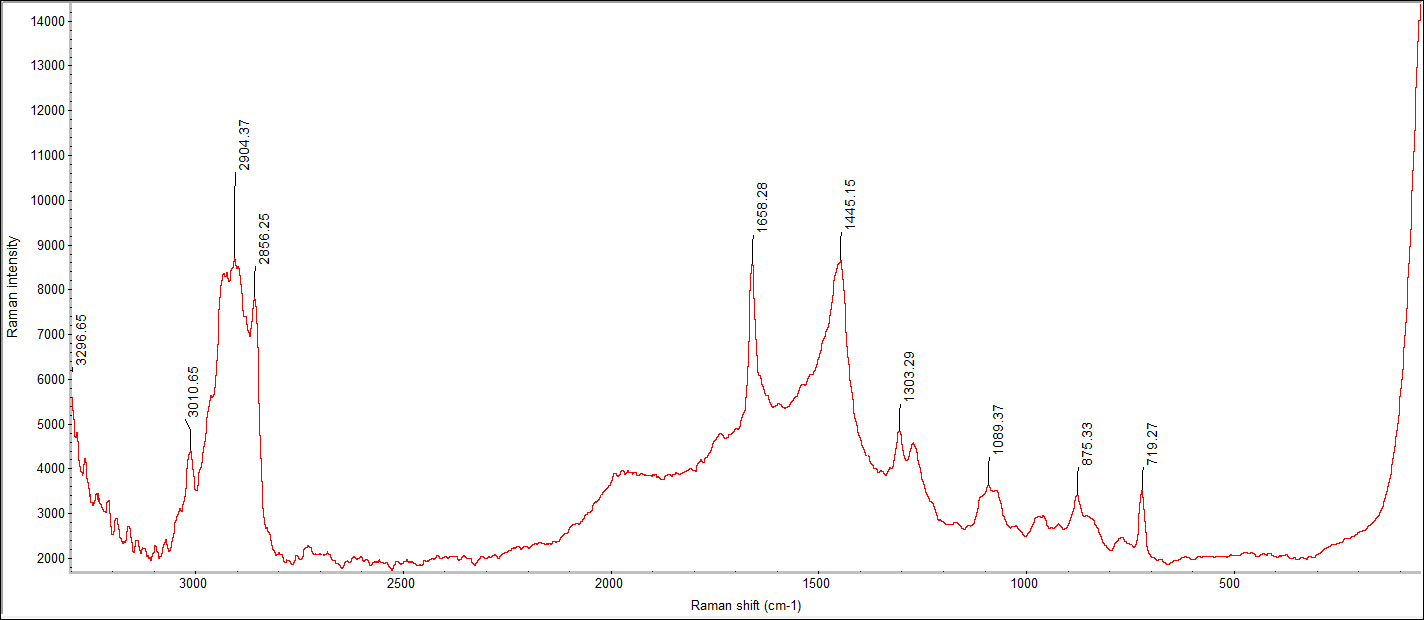
**

**DSC Spectra**

**DSC spectra of Flurbiprofen**

**
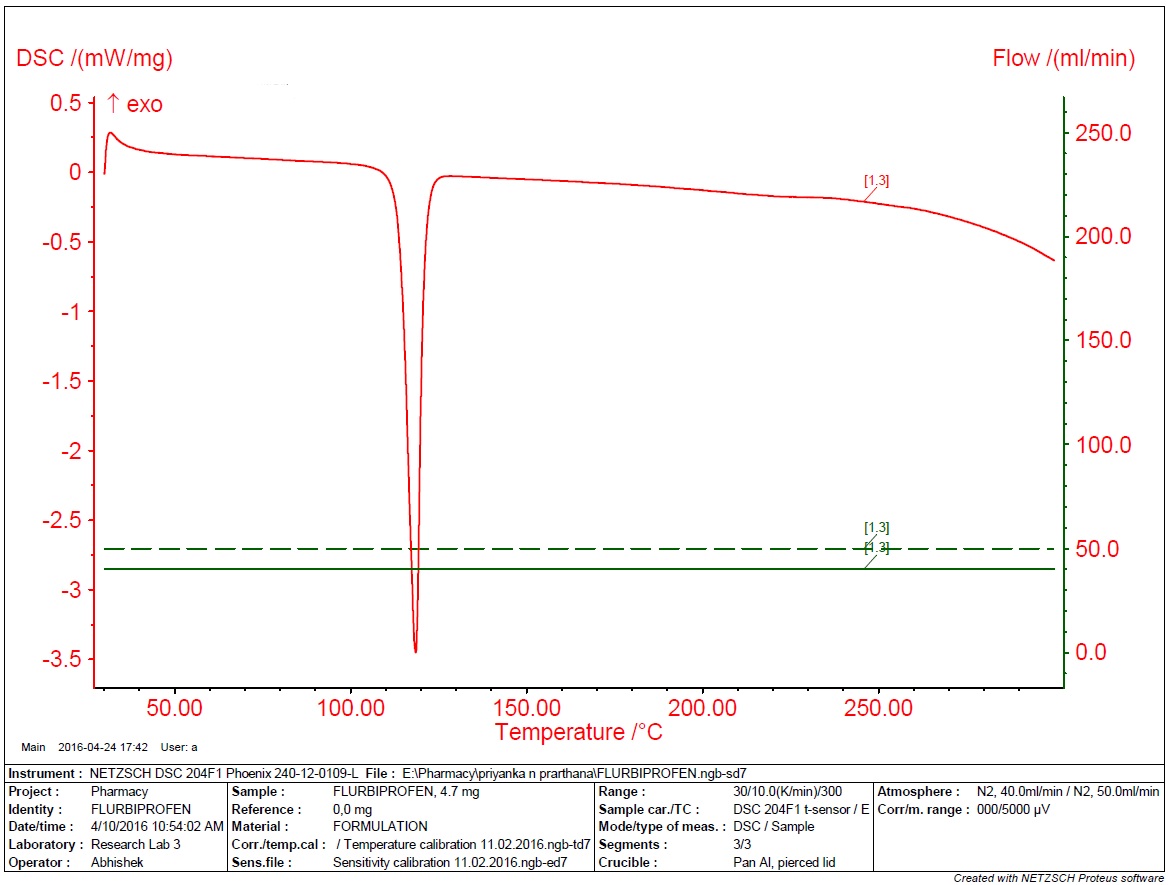
**

**DSC spectra of soyalecithin**


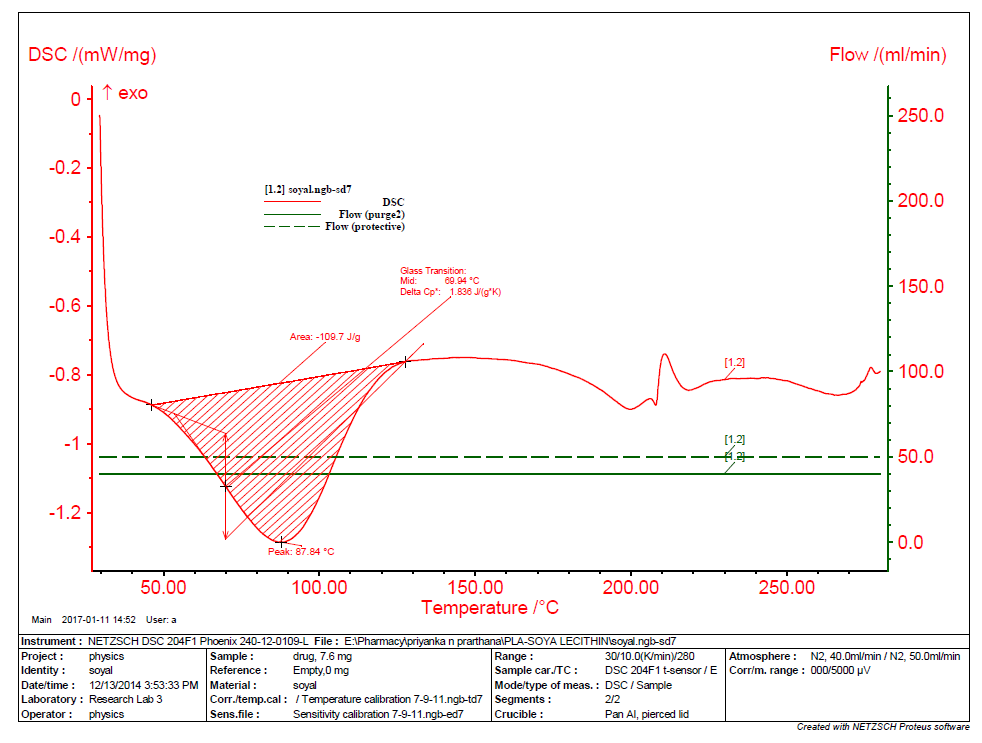


**DSC spectra of optimized formulation**

**
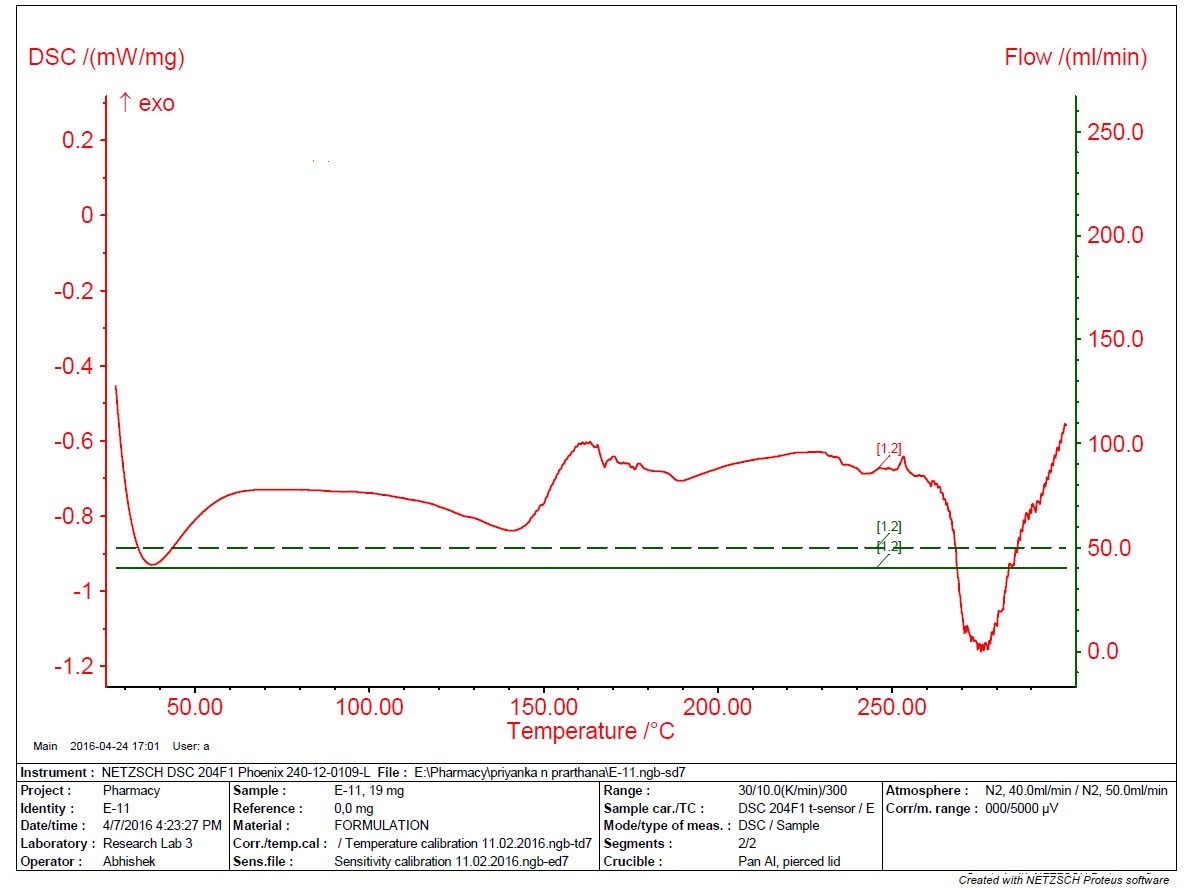
**

**TGA spectra**

**TGA spectra of flurbiprofen**

**
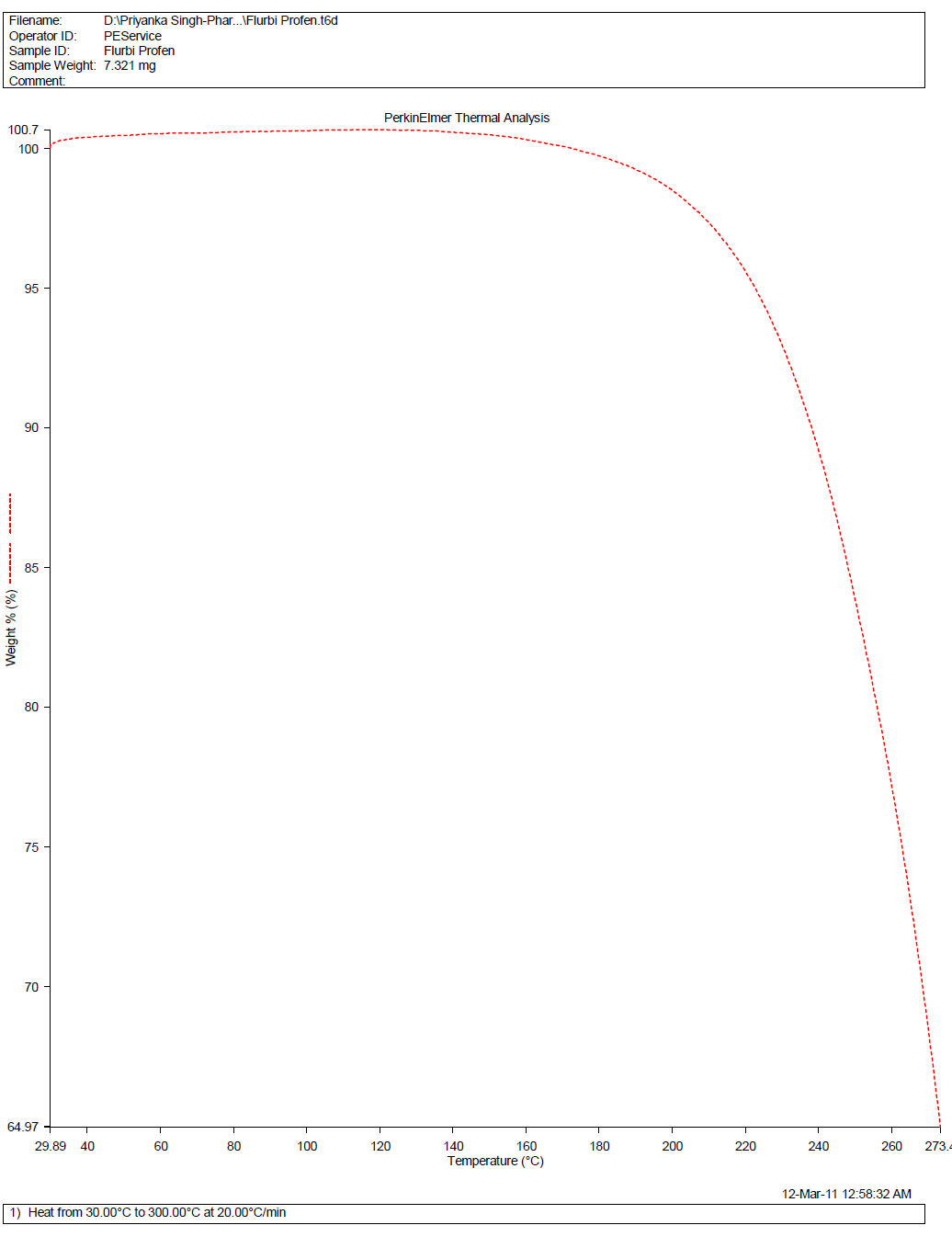
**

**TGA spectra of soyalecithin**

**
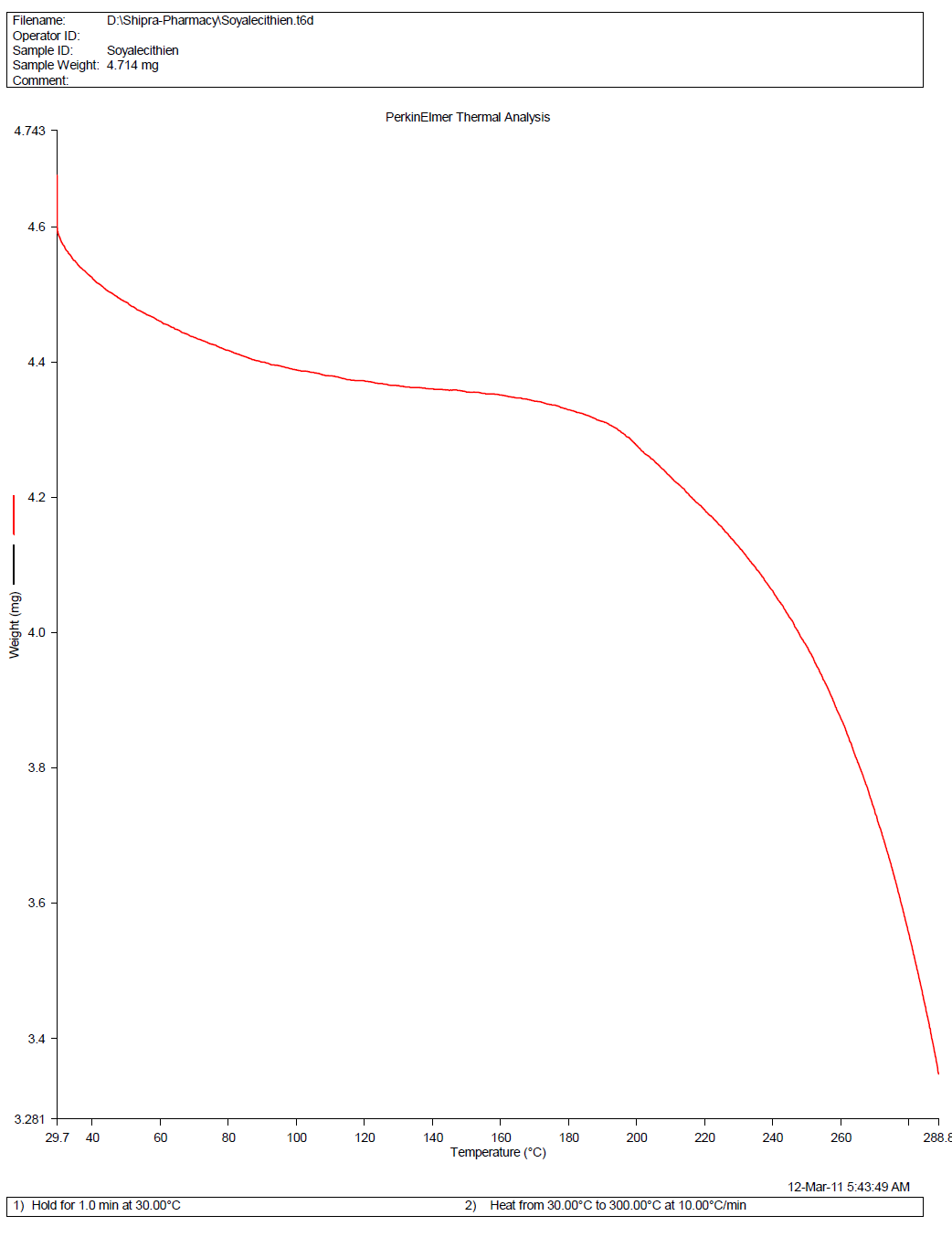
**

**TGA spectra of optimized formulation**

**
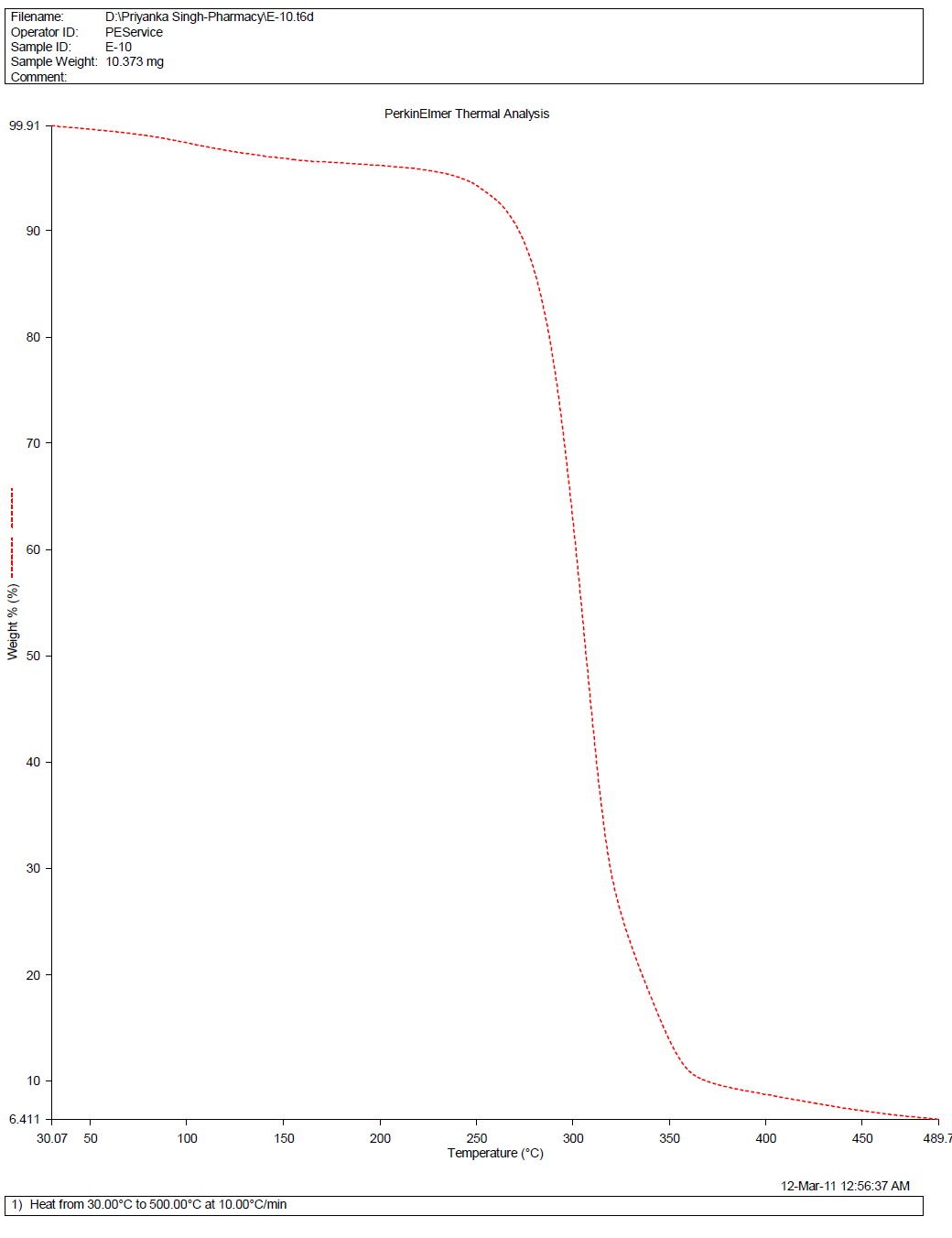
**

**% entrapment efficiency**

**
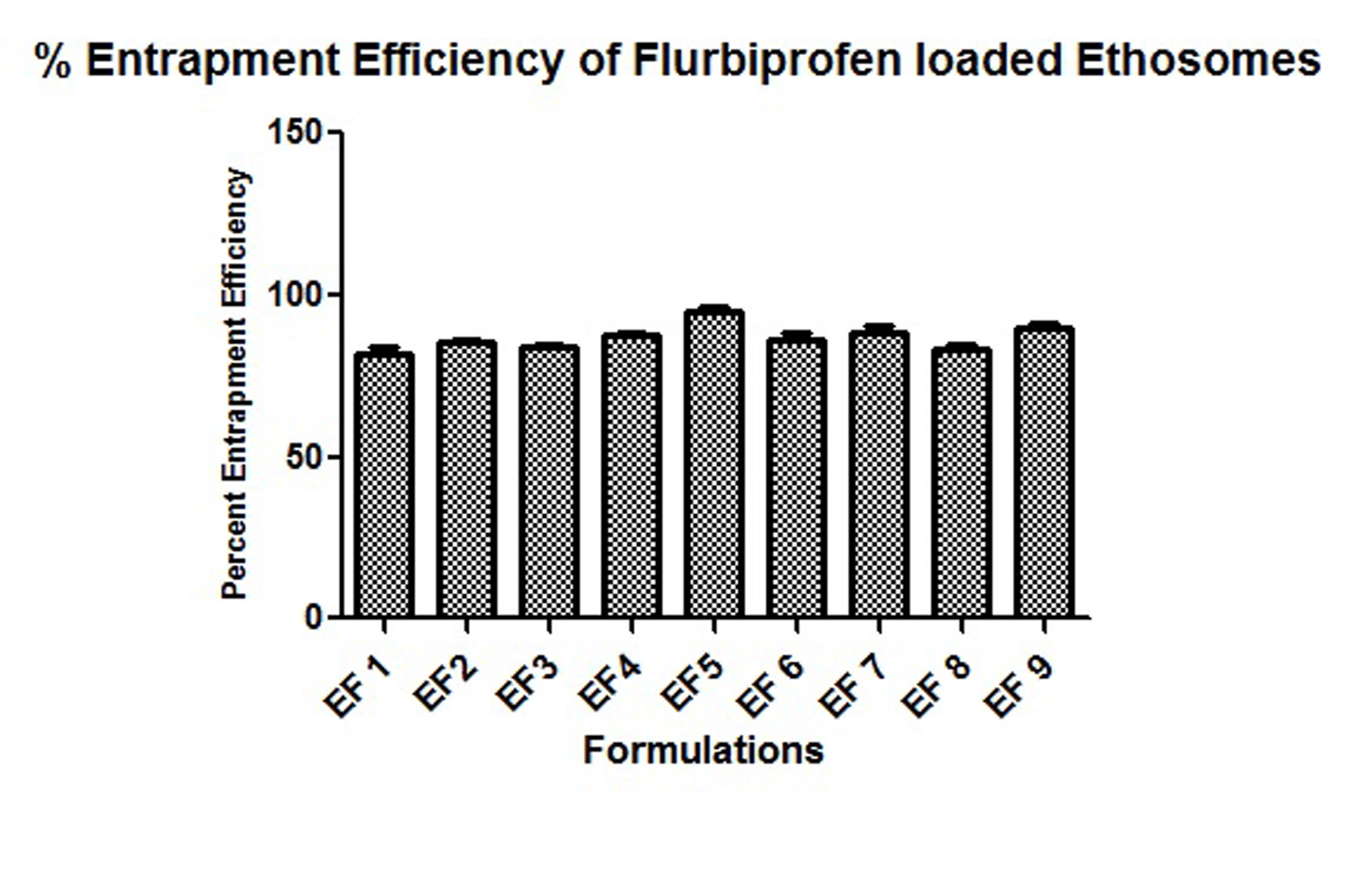
**

**Table 1-**Screening of analgesic activity of selected formulations using cold plate test in rats

| **S.No.** | **Treatment** | **Reaction time (sec.)** | | | |
| --- | --- | --- | --- | --- | --- |
|  |  | **15 min** | **30 min** | **45 min** | **60 min** |
| I | Brugel Gel | 16.9±2.02 | 17.7±2.4 | 19±2.21 | 19.8±2.41 |
| II | Etho 5 (10mg/kg) | 15.33±0.51 ^ns^ | 16.8±0.63 ^ns^ | 19.2±0.63 ^ns^ | 21±0.63 ^ns^ |
| III | Etho 5 (20mg/kg) | 15.66±0.81 ^ns^ | 17.48±0.5 ^ns^ | 21.66±0.51^**^ | 25.16±0.40^***^ |

**Table 2- Screening of anti-inflammatory activity of selected formulation** in carrageenan induced paw edema in rats

| **S.No.** | **Treatment** | **Swelling index (cm)** | | | | | |
| --- | --- | --- | --- | --- | --- | --- | --- |
|  |  | **15 min** | **30 min** | **45min** | **60 min** | **75 min** | **90 min** |
| I | Brugel Gel | 2.81±0.04 | 2.76±0.05 | 2.68±0.04 | 2.63±0.05 | 2.59±0.06 | 2.55±0.05 |
| II | Etho5 (10mg/kg) | 2.83±0.05 ^ns^ | 2.7±0.06 ^ns^ | 2.66±0.05 ^ns^ | 2.53±0.05^***^ | 2.45±0.05^***^ | 2.41±0.04^***^ |
| III | Etho5 (20mg/kg) | 2.82±0.06^ns^ | 2.67±0.05^***^ | 2.62±0.06 ^ns^ | 2.55±0.05 ^ns^ | 2.45±0.05^***^ | 2.35±0.05^***^ |
